# Supplementary material for: Full-Length Transcriptome Maps of Reef-Building Coral Illuminate the Molecular Basis of Calcification, Symbiosis, and Circadian Genes
Source: Int J Mol Sci. 2022 Sep 22;23(19):11135. doi: 10.3390/ijms231911135 (PMC9570262; doi:10.3390/ijms231911135)
Supplement: Supplementary file 1 [file ijms-23-11135-s001.zip › Supplementary figures_Figures S1-S38.pdf]

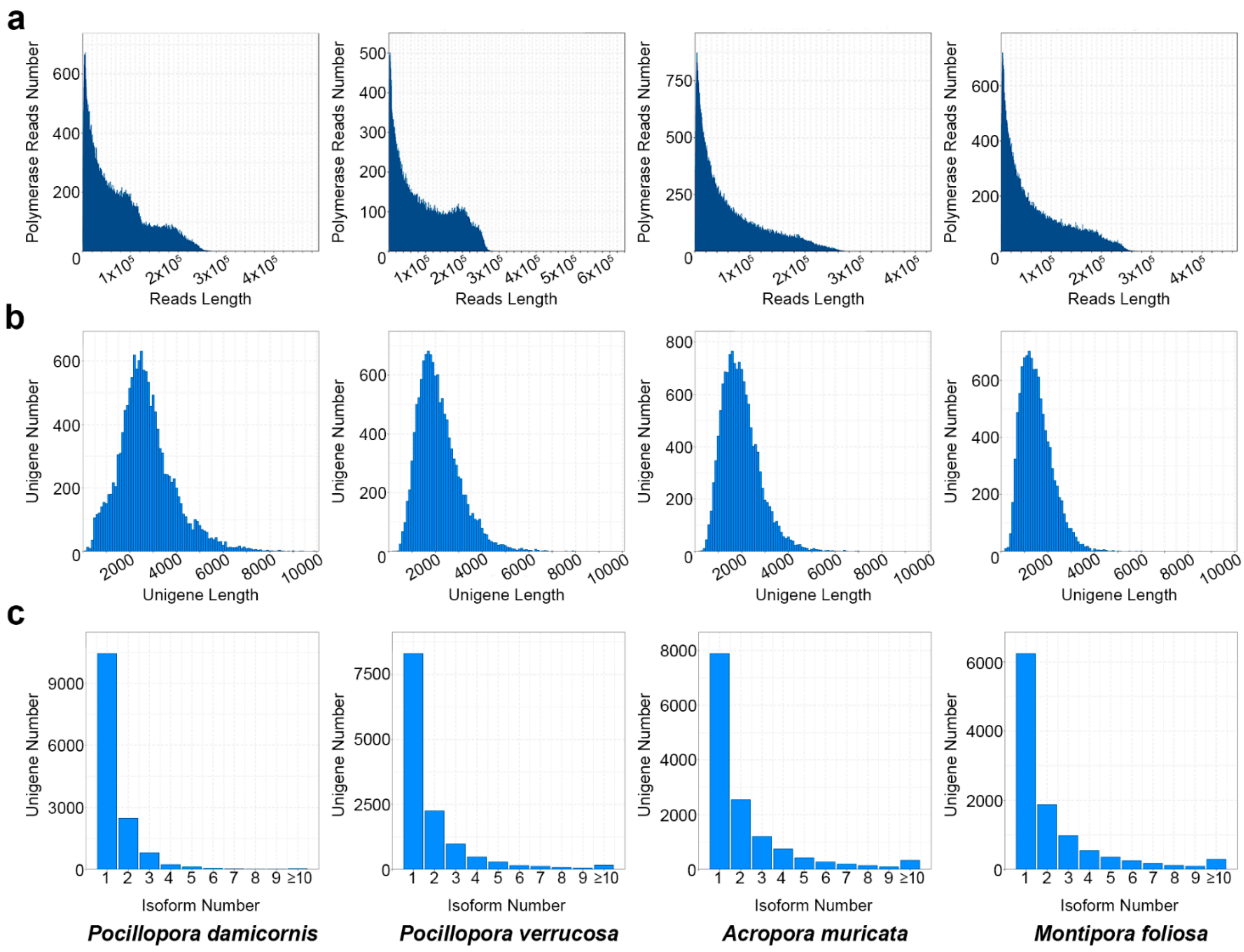

Figure S1. Overview of PacBio Sequel II SMRT sequencing data processing. **a.** Polymerase reads length distribution. The horizontal axis represents reads length, the vertical axis represents the number of different read lengths. **b.** Unigene length distribution. The horizontal axis represents unigene length, the vertical axis represents the number of different unigene lengths. **c.** Correspondence between unigene and isoform. The horizontal axis represents isoform number, the vertical axis represents the number of unigenes containing the same number of isoforms.

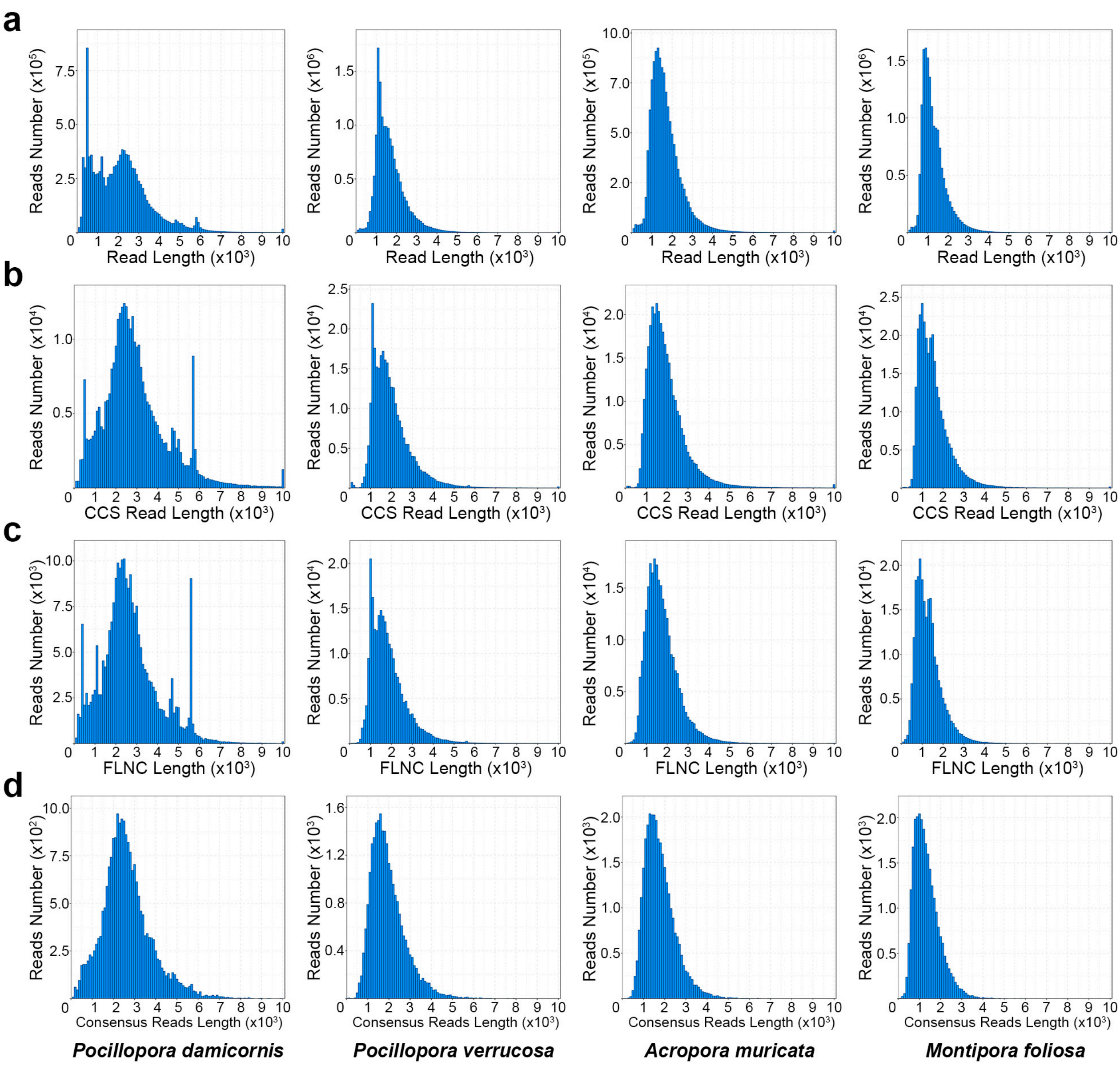

Figure S2. Intermediate processes of PacBio Sequel II SMRT sequencing data processing. **a.** Subreads length distribution. The horizontal axis represents reads length, the vertical axis represents the number of different read lengths. **b.** CCS reads length distribution. The horizontal axis represents CCS reads length, the vertical axis represents the number of different read lengths. **c.** Full-Length non-chimericRead (FLNC) length distribution. The horizontal axis represents FLNC length, the vertical axis represents the number of different read lengths. **d.** Polished consensus reads length distribution. The horizontal axis represents reads length, the vertical axis represents the number of different read lengths.

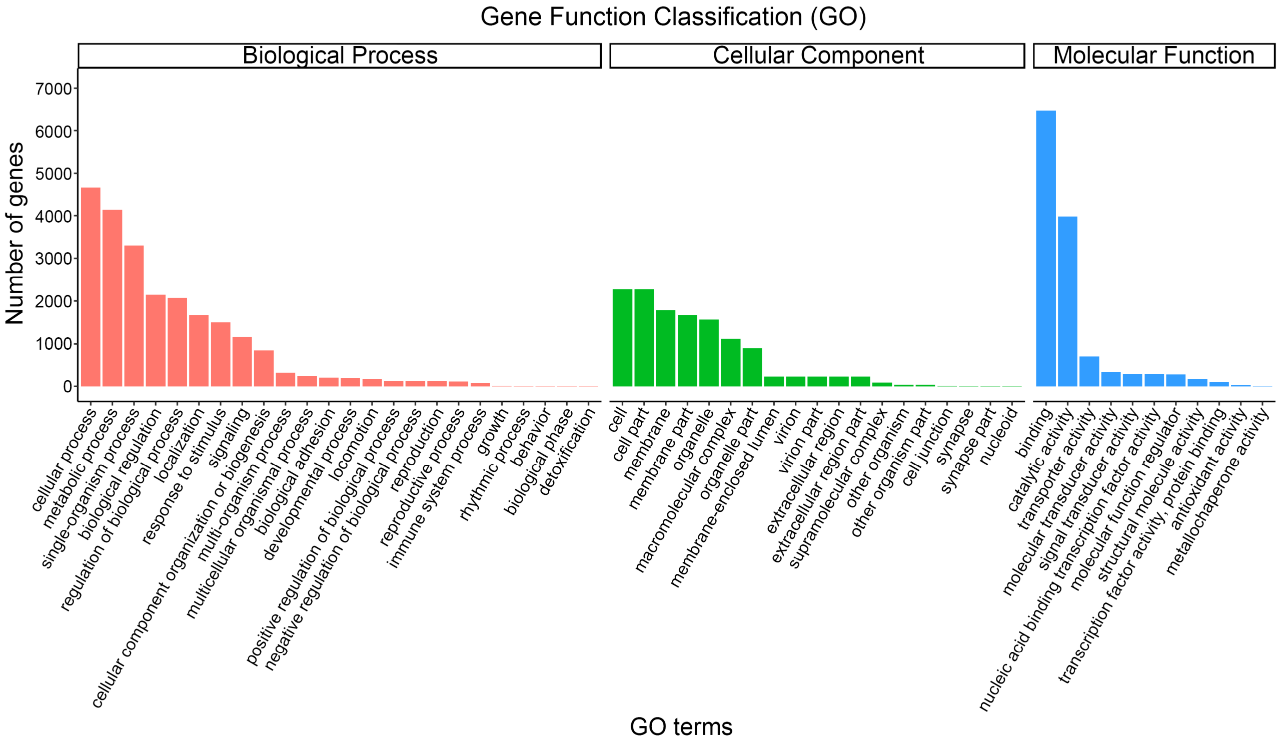

Figure S3. GO classifications of coral genes in *P. damicornis*. The horizontal axis represents the GO terms at the next level of the three major GO categories, the vertical axis represents the number of genes annotated to the term (including subterms of the term). Three different categories represent the three basic classifications of Go terms (from left to right, biological processes, cellular components, and molecular functions).

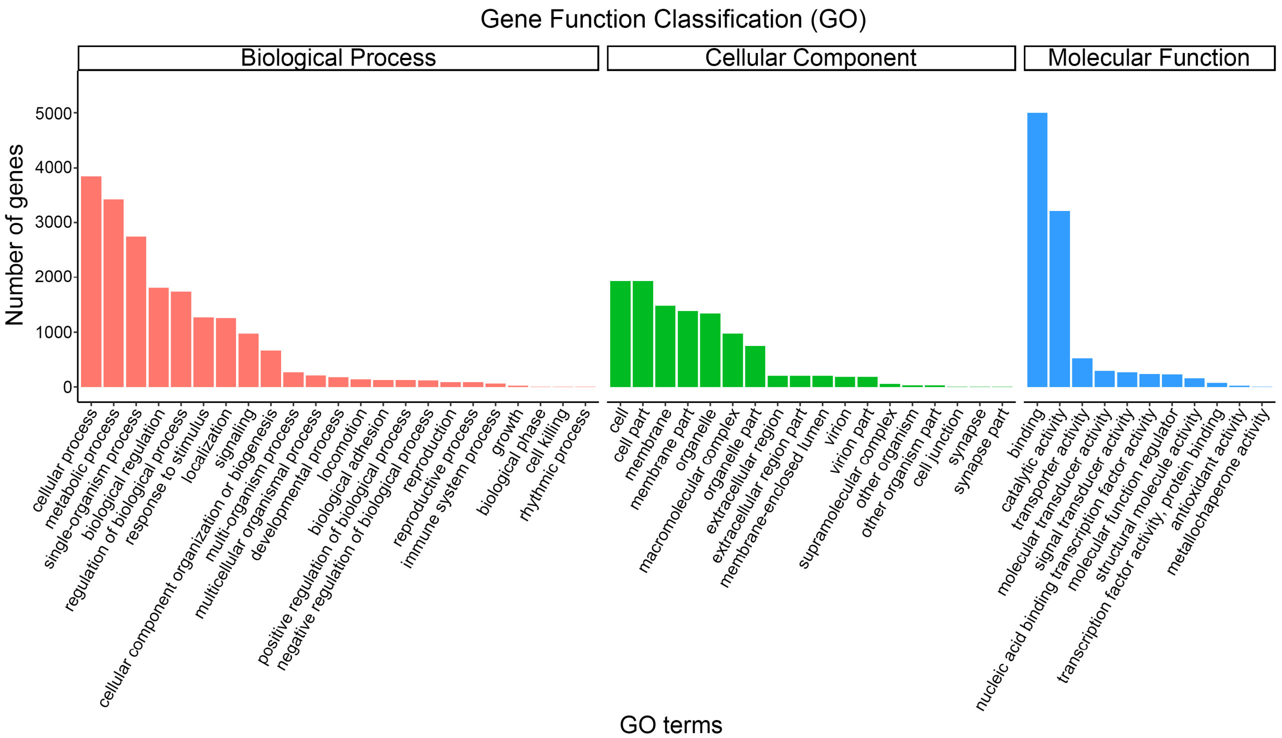

Figure S4. GO classifications of coral genes in *P. verrucosa*. The horizontal axis represents the GO terms at the next level of the three major GO categories, the vertical axis represents the number of genes annotated to the term (including subterms of the term). Three different categories represent the three basic classifications of Go terms (from left to right, biological processes, cellular components, and molecular functions).

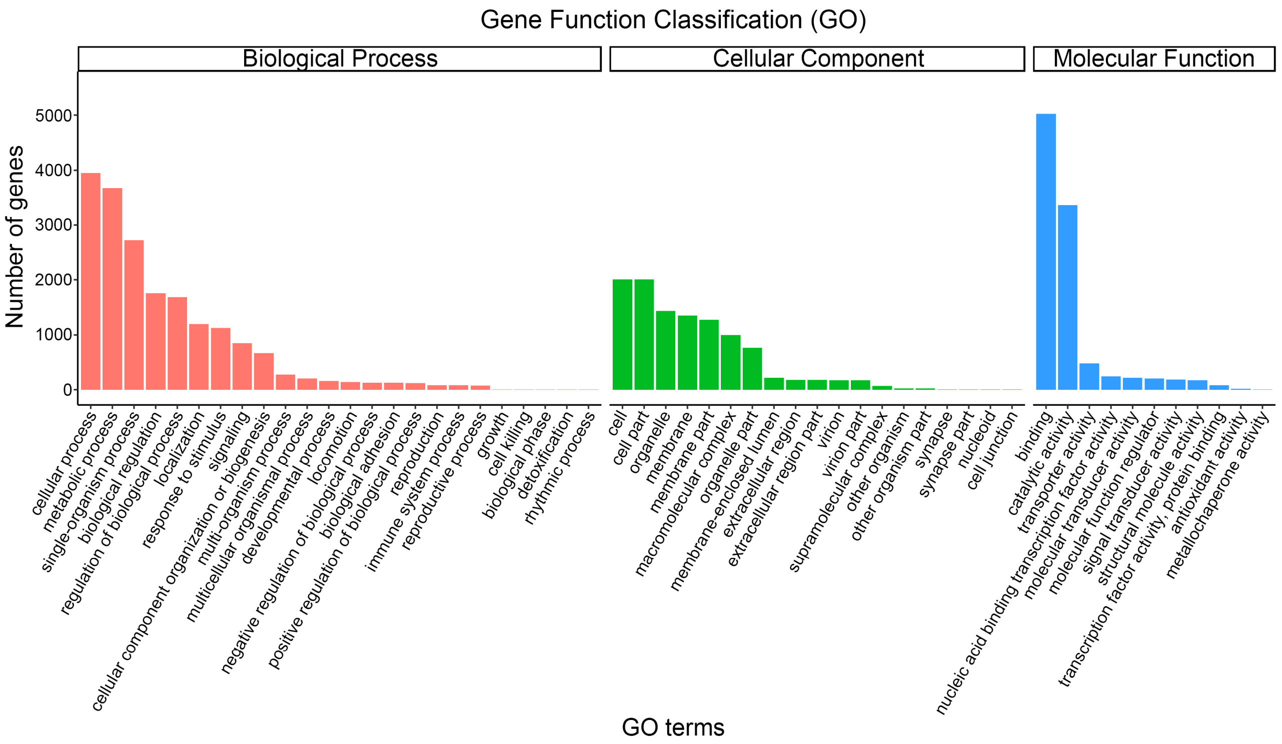

Figure S5. GO classifications of coral genes in *A. muricata*. The horizontal axis represents the GO terms at the next level of the three major GO categories, the vertical axis represents the number of genes annotated to the term (including subterms of the term). Three different categories represent the three basic classifications of Go terms (from left to right, biological processes, cellular components, and molecular functions).

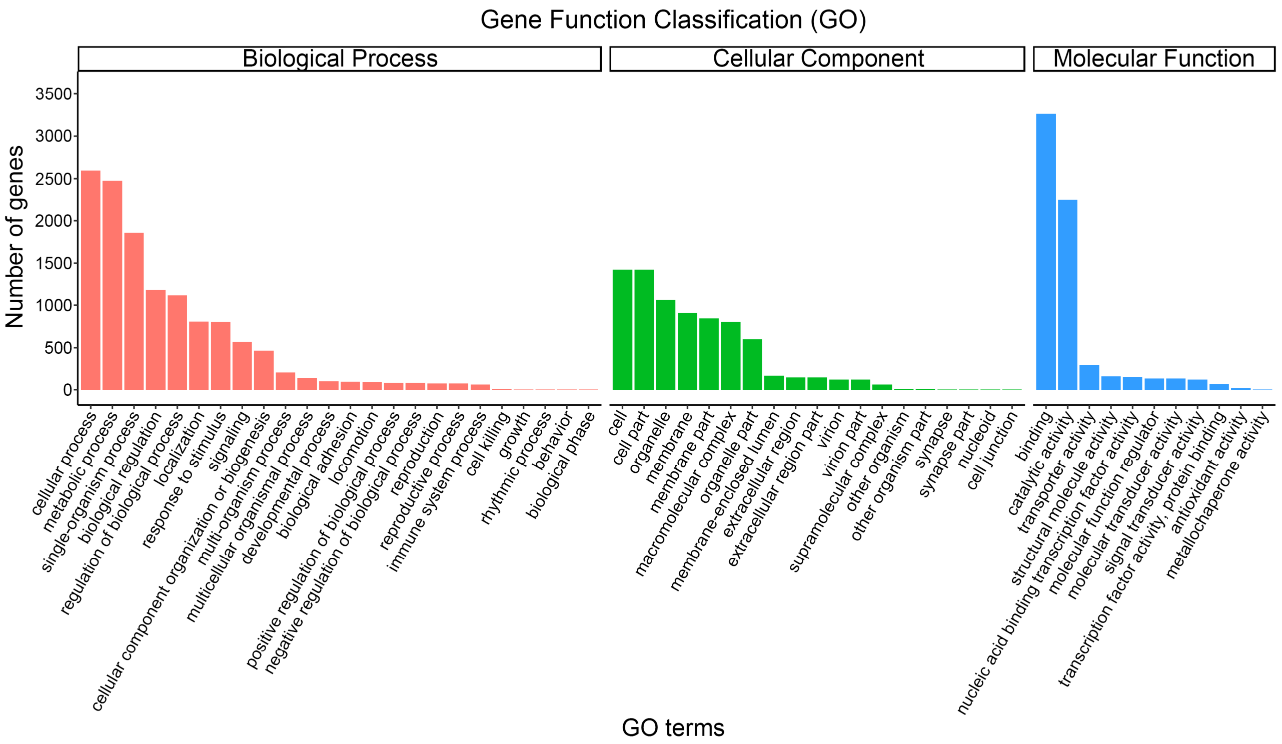

Figure S6. GO classifications of coral genes in *M. foliosa*. The horizontal axis represents the GO terms at the next level of the three major GO categories, the vertical axis represents the number of genes annotated to the term (including subterms of the term). Three different categories represent the three basic classifications of Go terms (from left to right, biological processes, cellular components, and molecular functions).

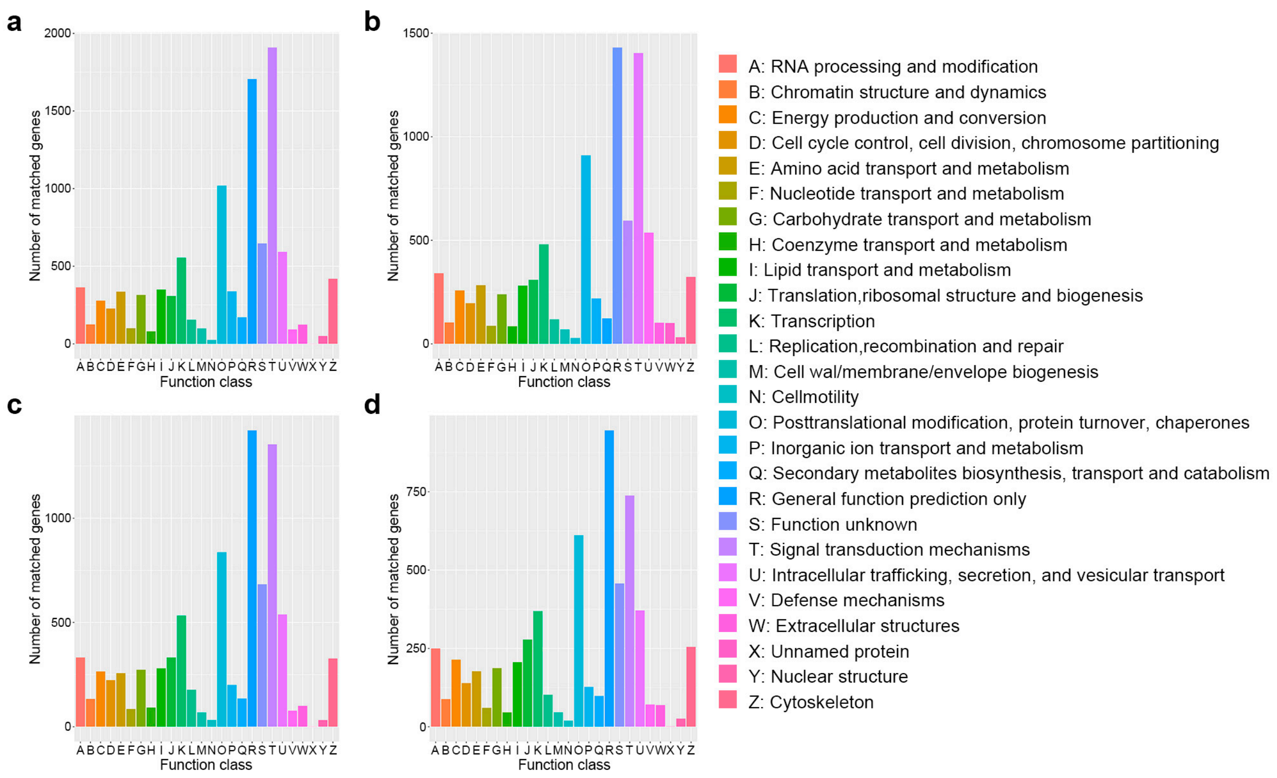

Figure S7. KOG classifications of coral genes. **a-d** represent *P. damicornis, P. verrucosa, A. muricata* and *M. foliosa* respectively. The horizontal axis is the name of the 26 function classes of KOG/COG, and the vertical axis is the number of genes matched to different classes.

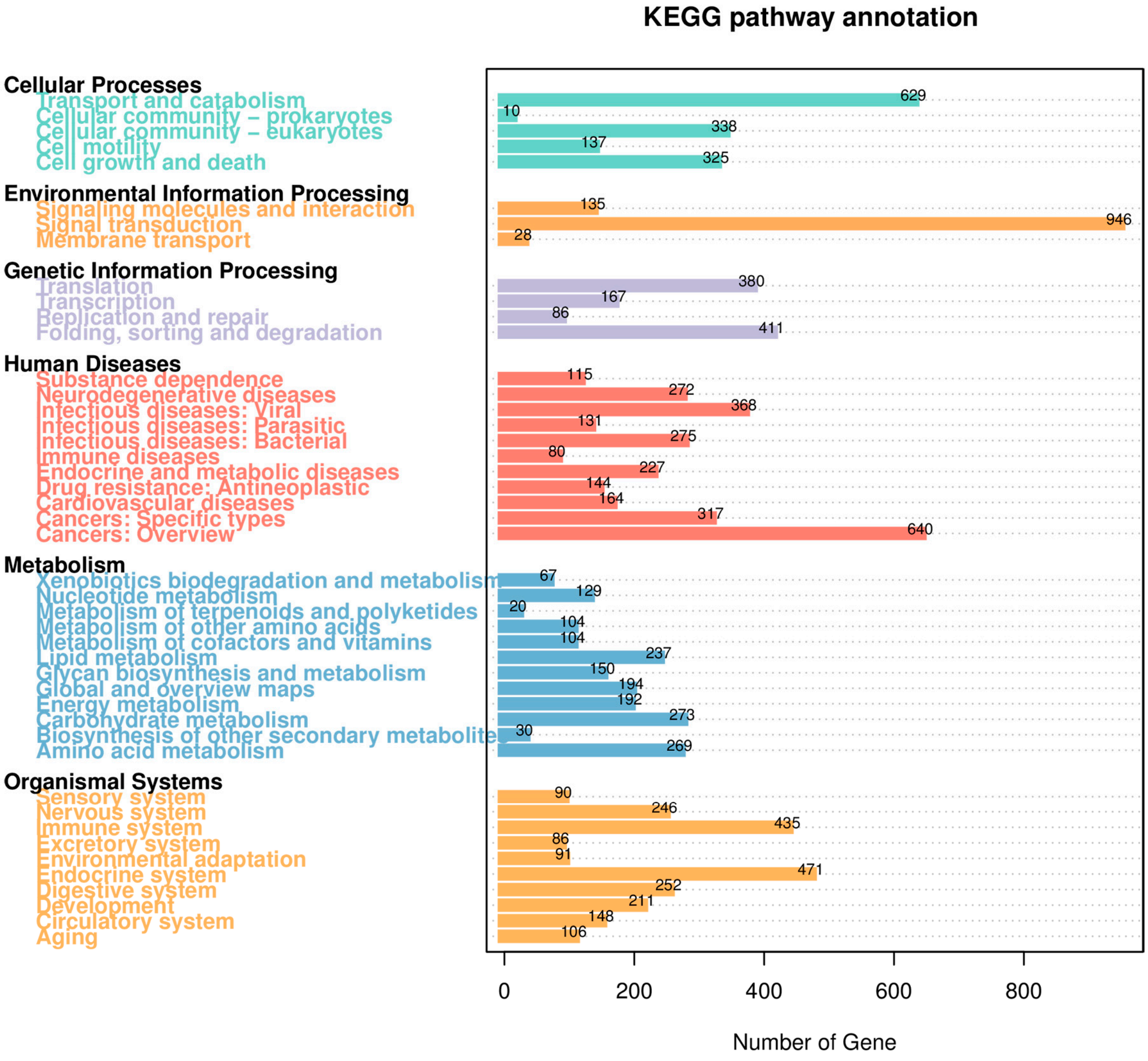

Figure S8. KEGG metabolic pathway classifications of coral genes in *P. damicornis*. On the left are the different KEGG categories, including cellular processes, environmental information processing, genetic information processing, human diseases, metabolism and organismal systems. On the right is the number of their corresponding genes.

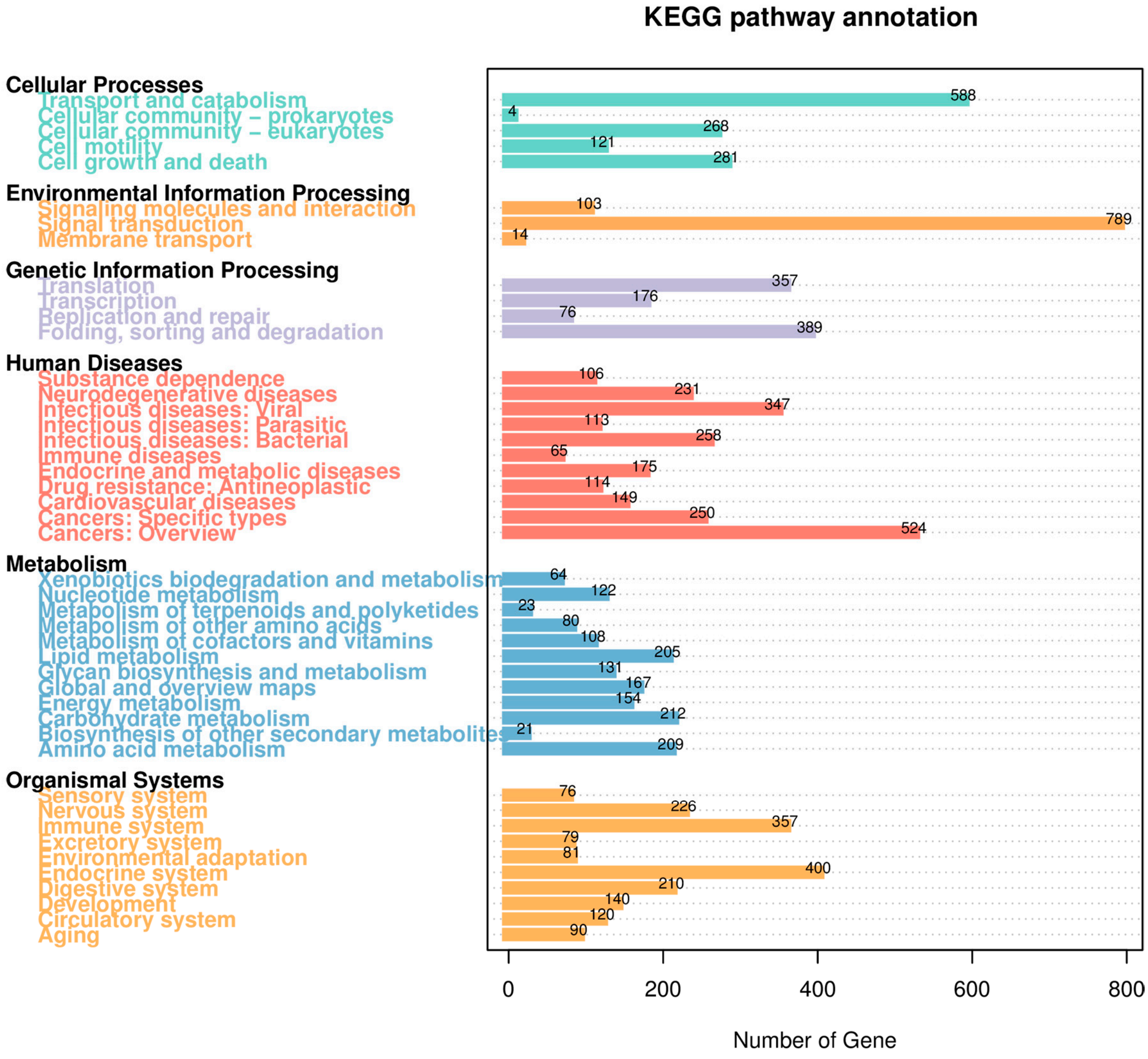

Figure S9. KEGG metabolic pathway classifications of coral genes in *P. verrucosa*. On the left are the different KEGG categories, including cellular processes, environmental information processing, genetic information processing, human diseases, metabolism and organismal systems. On the right is the number of their corresponding genes.

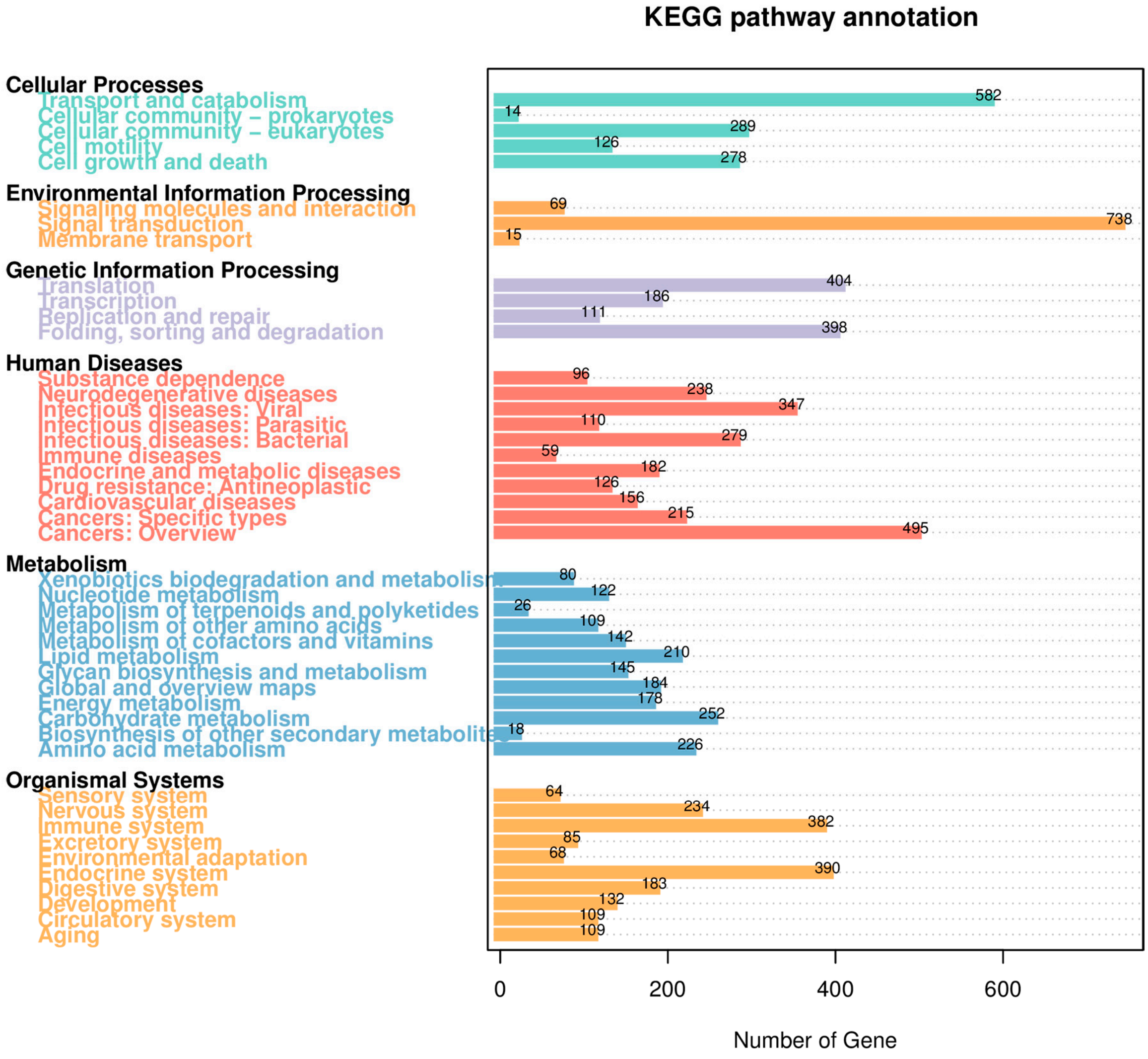

Figure S10. KEGG metabolic pathway classifications of coral genes in *A. muricata*. On the left are the different KEGG categories, including cellular processes, environmental information processing, genetic information processing, human diseases, metabolism and organismal systems. On the right is the number of their corresponding genes.

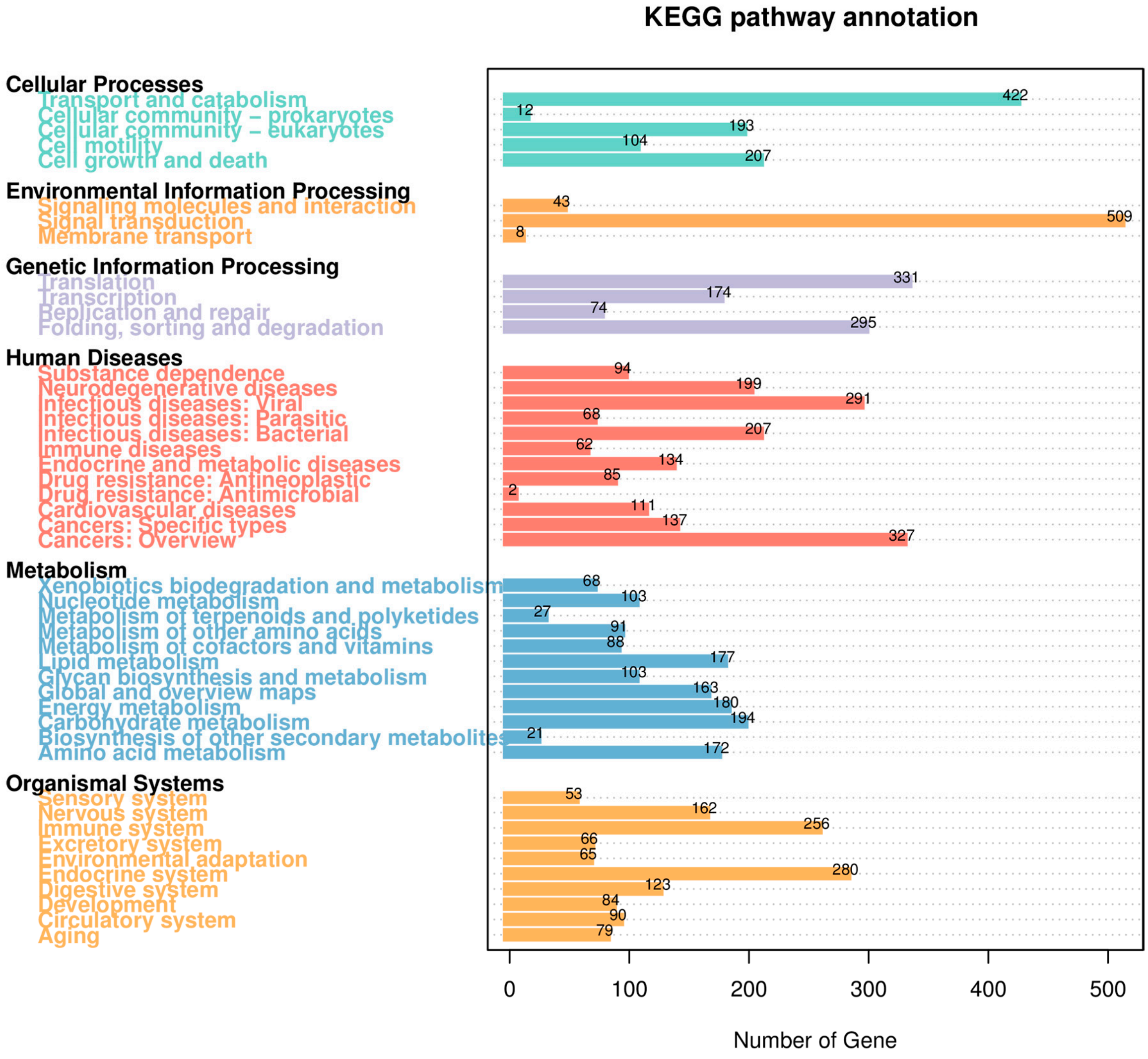

Figure S11. KEGG metabolic pathway classifications of coral genes in *M. foliosa*. On the left are the different KEGG categories, including cellular processes, environmental information processing, genetic information processing, human diseases, metabolism and organismal systems. On the right is the number of their corresponding genes.

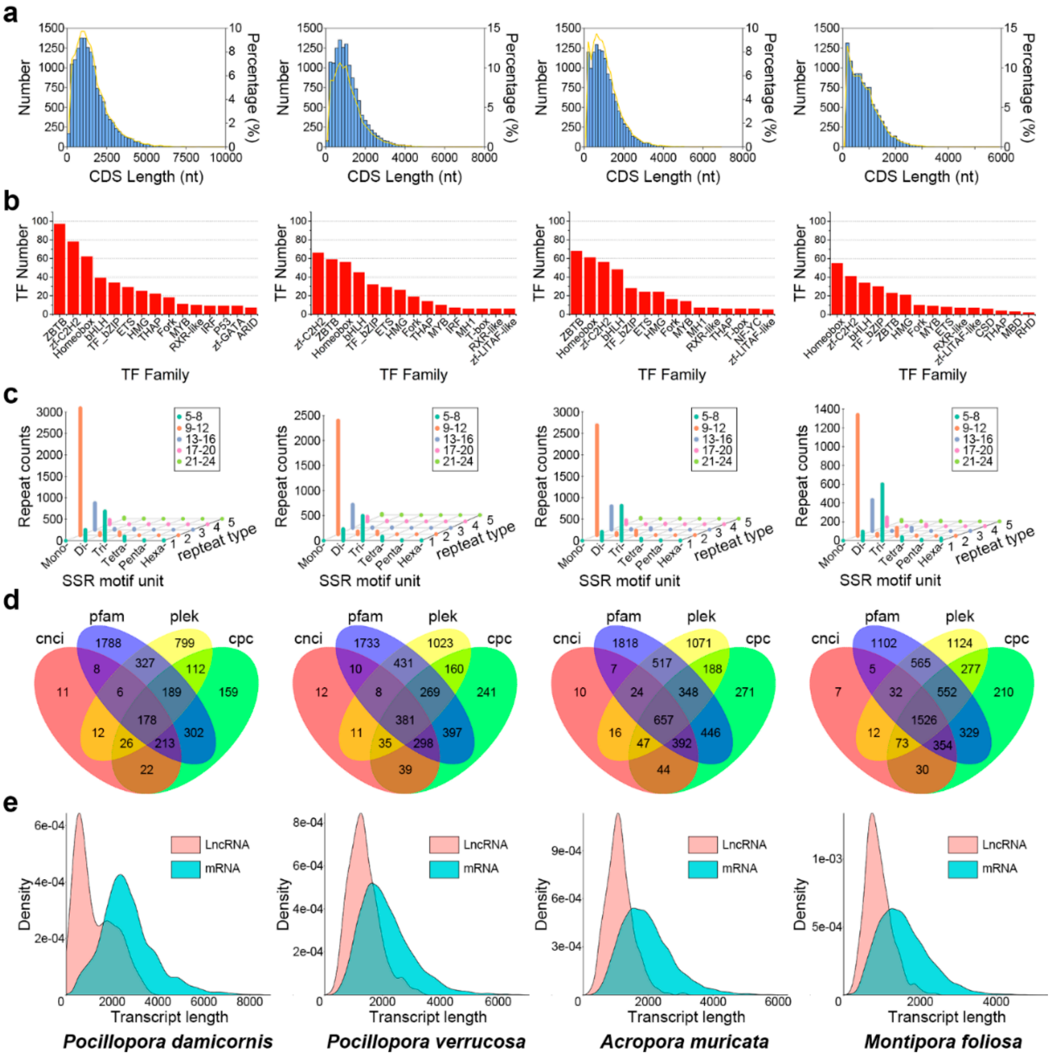

Figure S12. Summary of coral gene structural analysis. **a.** CDS length distribution. The horizontal axis represents the length of the predicted CDS, the vertical axis represents the number (blue bar chart) and percentage (yellow curve chart) of transcripts of the CDS. **b**. Predicted TF family. The horizontal axis represents the top 15 predicted transcription factor families in coral, and the vertical axis represents the number of them. **c**. SSR motif distribution. The X coordinate (SSR motif unit) is the SSR type, which refers to the number of repeating bases, the Y coordinate (repeat type) is the number of base (SSR motif unit) repeats, using different colors mean different repeat number intervals (the details are seen in the legend), the Z coordinate (Repeat counts) is the number of SSRs. **d**. Venn plots of predicted lncRNA. The sum of the numbers in each large circle represents the number of lncRNA predicted by one of CNCI, PLEK, CPC2 and Pfam databases, and the overlapping circles indicate the number of lncRNA predicted by these two or more databases simultaneously. **e**. LncRNA and mRNA length distribution comparison. The horizontal axis is the length of transcripts, the vertical axis is their density of distribution.

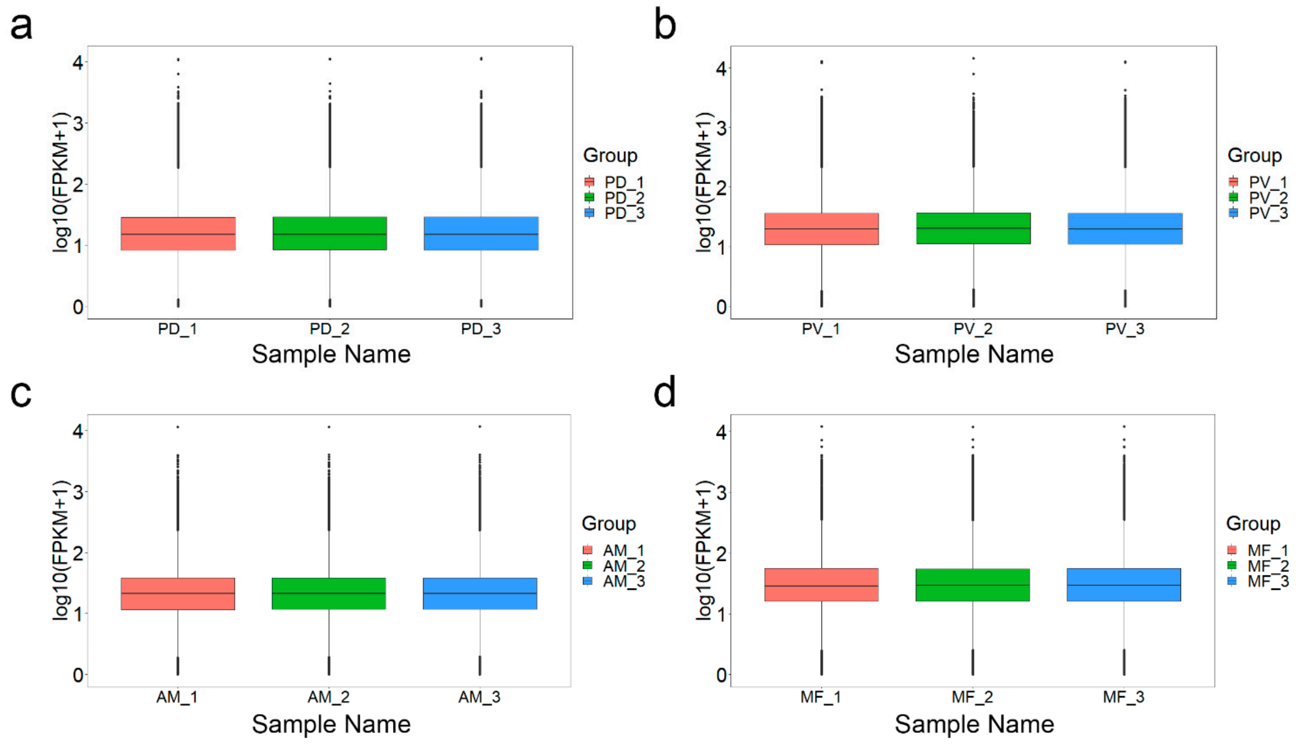

Figure S13. FPKM box plot of each coral gene expression. The horizontal axis is the sample name, *P. damicornis* (**a**), *P. verrucosa* (**b**), *A. muricata* (**c**) and *M. foliosa* (**d**), the vertical axis is log10 (FPKM+1). Each box plot shows five statistics results, including maximum, upper quartile, median, lower quartile and minimum from top to down.

a

b

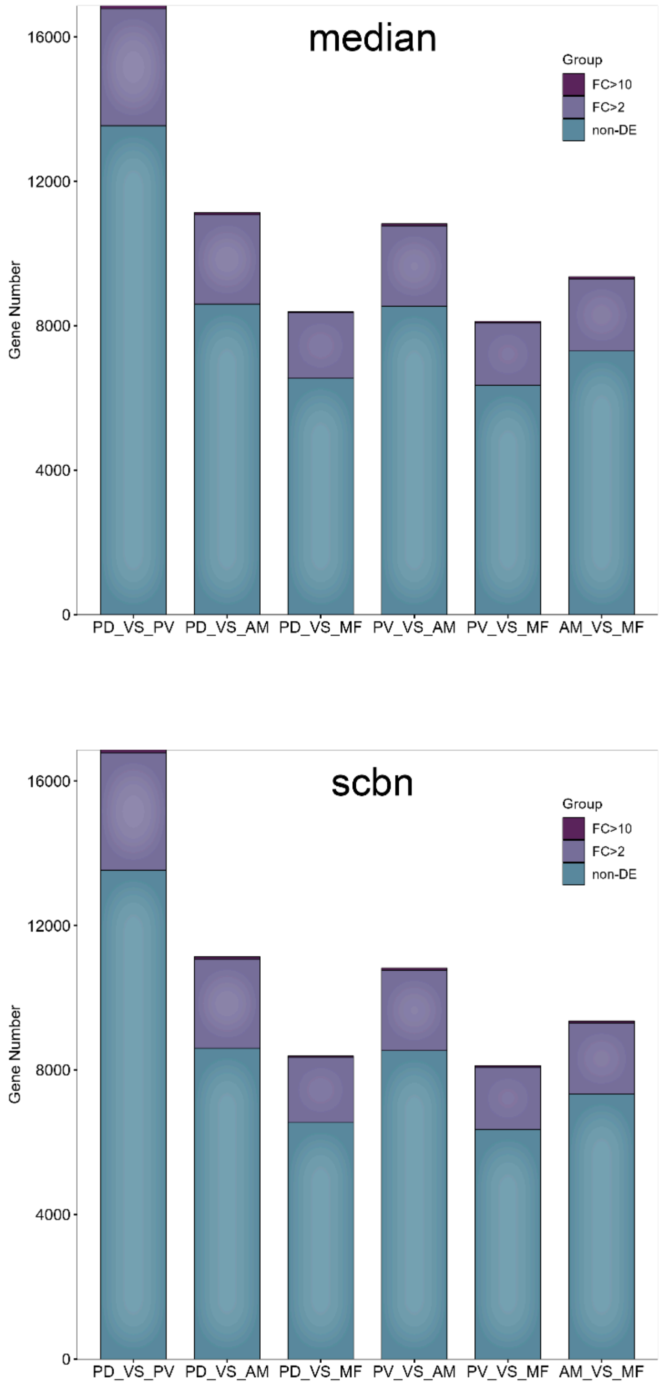

Figure S14. Number of DEGs (or transcript orthologs) between each two corals calculated by median (**a**) and scbn (**b**) methods. In the legend, "non-DE" represents orthologous transcripts based on the parameters |log2FoldChange|<=2 or p-value >=10^{-6}, "FC>2" represents orthologous transcripts based on the parameters 2<|log2FoldChange|<=10 and p-value <10^{-6}, and "FC>10" represents orthologous transcripts based on the parameters |log2FoldChange|>10 and p-value<10^{-6}. PD: *P. damicornis*; PV: *P. verrucosa*; AM: *A. muricata*; and MF: *M. foliosa*.

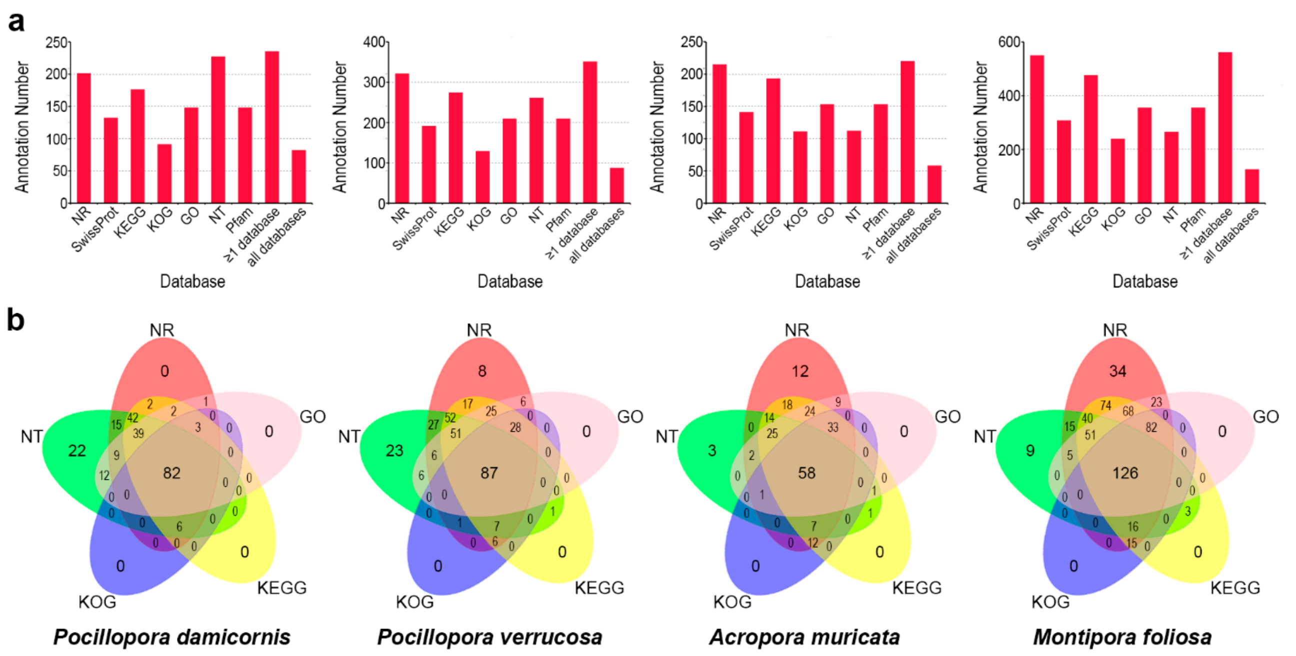

Figure S15. Summary of Symbiodiniaceae gene functional annotation. **a.** Statistics of the annotation results of Symbiodiniaceae in four reef-building corals, in the NR, Swiss-Prot, KEGG, KOG, GO NT and Pfam databases. The horizontal axis represents the different functional databases, and the vertical axis represents the number of sequences annotated in different functional databases, at least one database and all databases. **b.** Venn plots of the number of annotated sequences of Symbiodiniaceae in four reef-building corals obtained using the NR, KEGG, KOG, GO and NT databases. The sum of the numbers in each large circle represents the number of transcripts annotated in one database, and the overlapping circles indicate the number of transcripts annotated to these databases simultaneously.

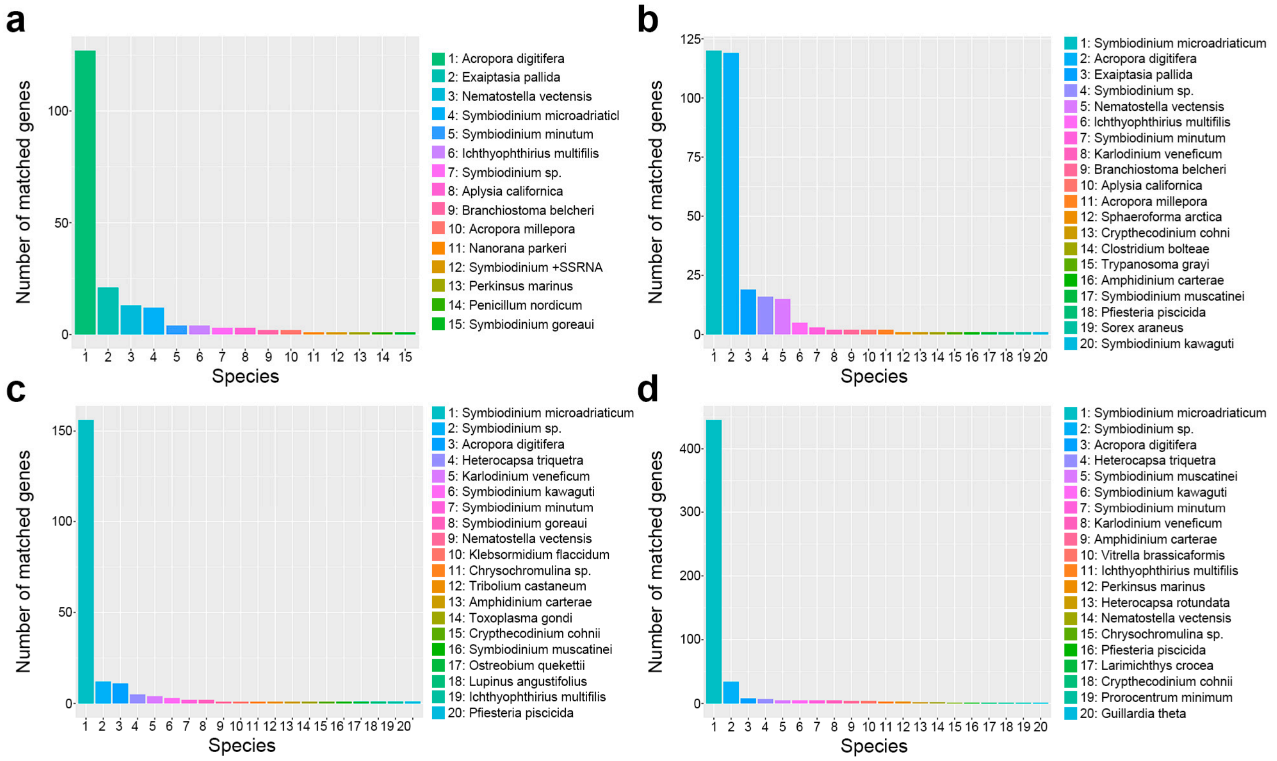

Figure S16. NR database annotation of Symbiodiniaceae. The top 20 species with the greatest number of top sequence hits to Symbiodiniaceae sequences of *P. damicornis* (**a**), *P. verrucosa* (**b**), *A. muricata* (**c**) and *M. foliosa* (**d**) are shown. The horizontal axis represents the species ID, and the vertical axis represents the number of Symbiodiniaceae unigenes annotated to different species.

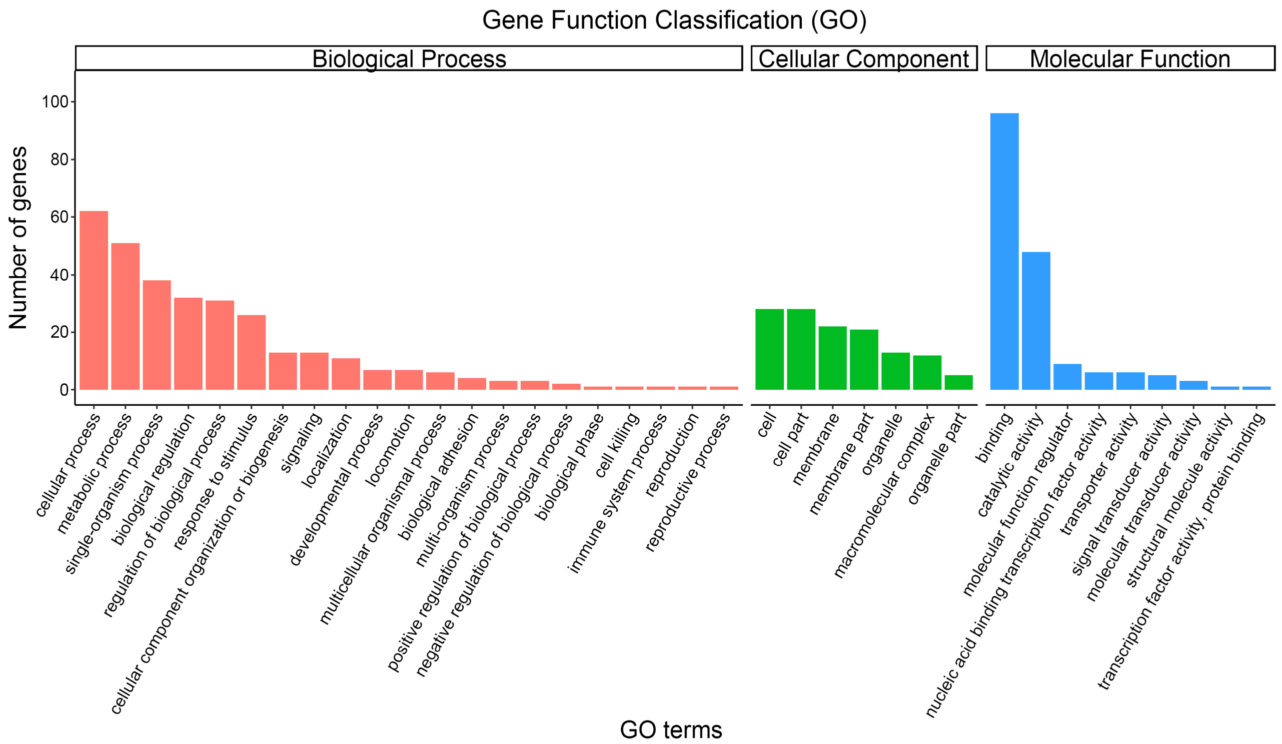

Figure S17. GO classifications of Symbiodiniaceae genes in *P. damicornis*. The horizontal axis represents the GO terms at the next level of the three major GO categories, the vertical axis represents the number of genes annotated to the term (including subterms of the term). Three different categories represent the three basic classifications of Go terms (from left to right, biological processes, cellular components, and molecular functions).

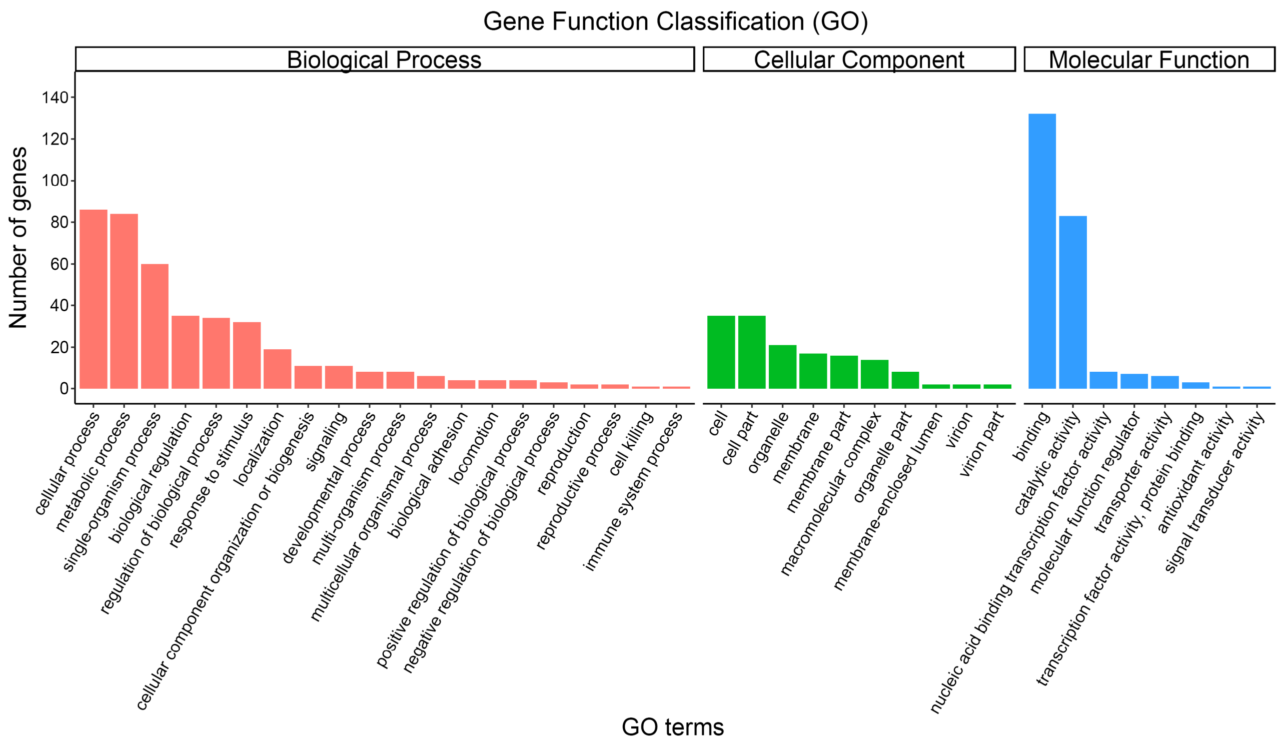

Figure S18. GO classifications of Symbiodiniaceae genes in *P. verrucosa*. The horizontal axis represents the GO terms at the next level of the three major GO categories, the vertical axis represents the number of genes annotated to the term (including subterms of the term). Three different categories represent the three basic classifications of Go terms (from left to right, biological processes, cellular components, and molecular functions).

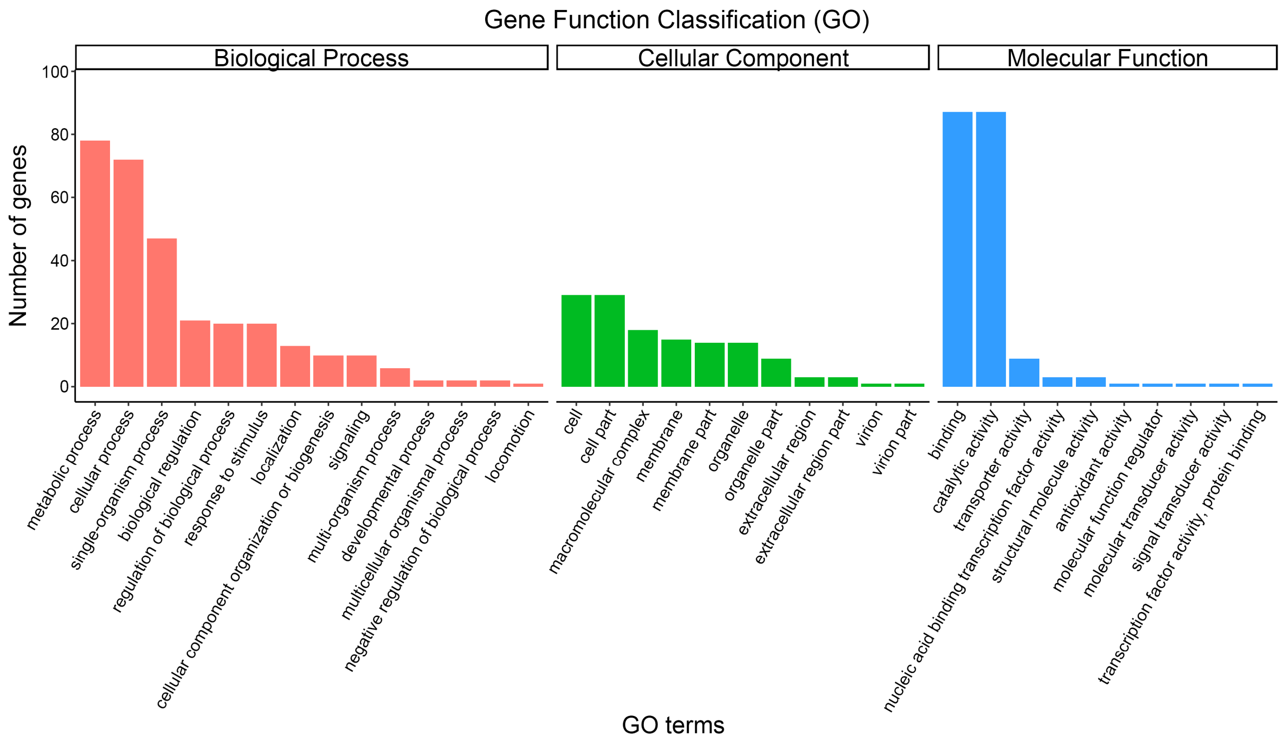

Figure S19. GO classifications of Symbiodiniaceae genes in *A. muricata*. The horizontal axis represents the GO terms at the next level of the three major GO categories, the vertical axis represents the number of genes annotated to the term (including subterms of the term). Three different categories represent the three basic classifications of Go terms (from left to right, biological processes, cellular components, and molecular functions).

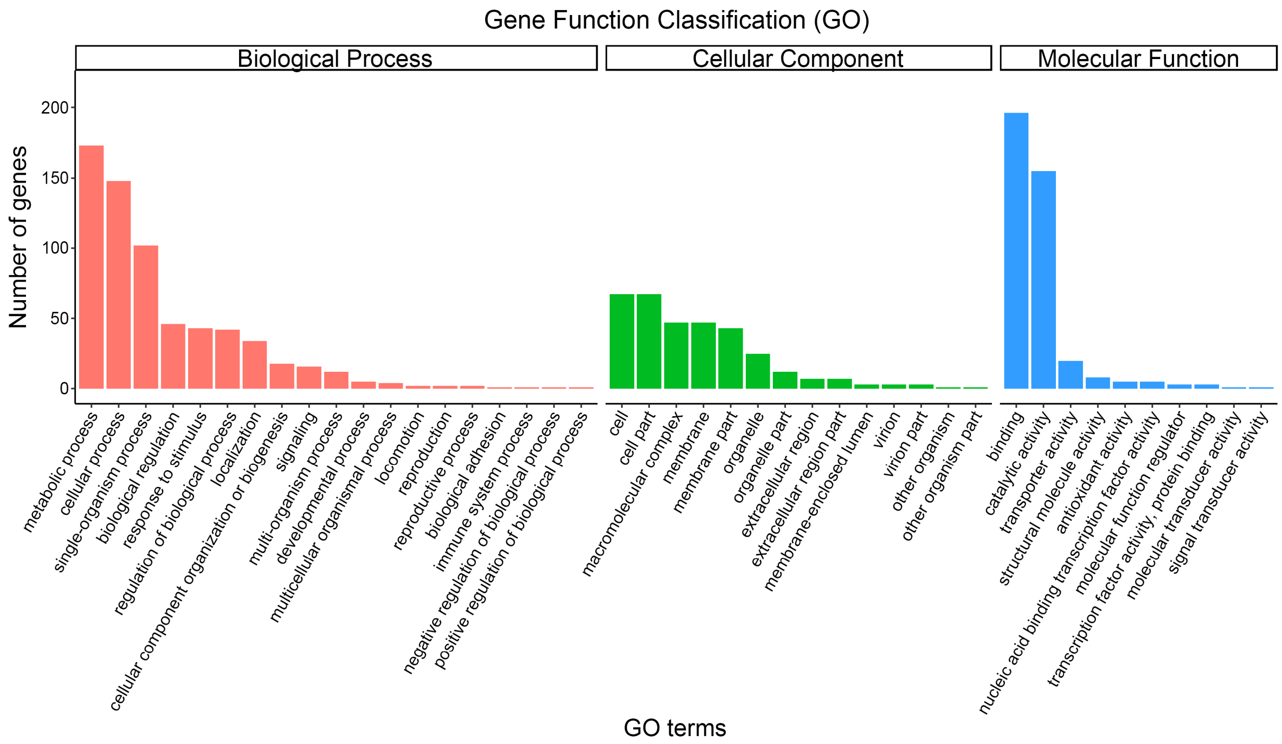

Figure S20. GO classifications of Symbiodiniaceae genes in *M. foliosa*. The horizontal axis represents the GO terms at the next level of the three major GO categories, the vertical axis represents the number of genes annotated to the term (including subterms of the term). Three different categories represent the three basic classifications of Go terms (from left to right, biological processes, cellular components, and molecular functions).

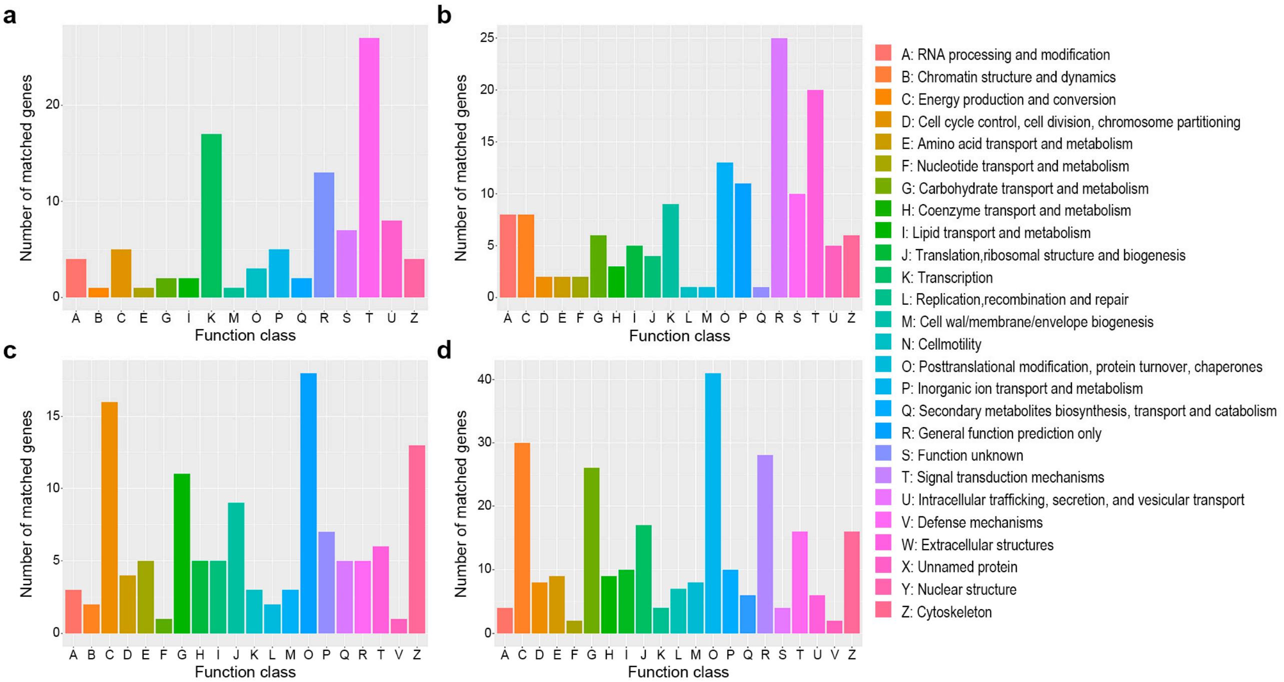

Figure S21. KOG classifications of Symbiodiniaceae genes. **a-d** represent *P. damicornis, P. verrucosa, A. muricata* and *M. foliosa* respectively. The horizontal axis is the name of the 26 function classes of KOG/COG, and the vertical axis is the number of genes matched to different classes.

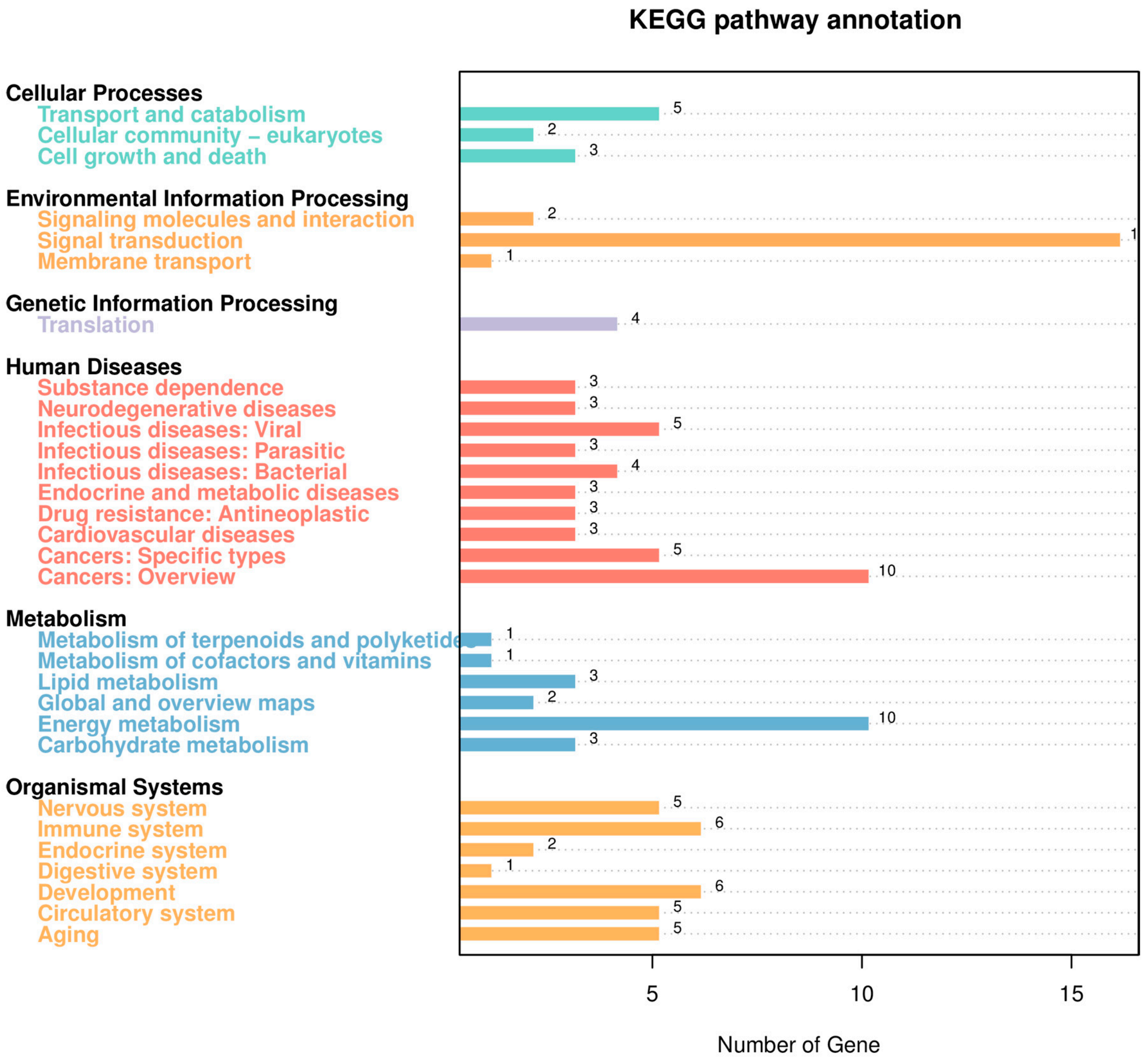

Figure S22. KEGG metabolic pathway classifications of Symbiodiniaceae genes in *P. damicornis*. On the left are the different KEGG categories, including cellular processes, environmental information processing, genetic information processing, human diseases, metabolism and organismal systems. On the right is the number of their corresponding genes.

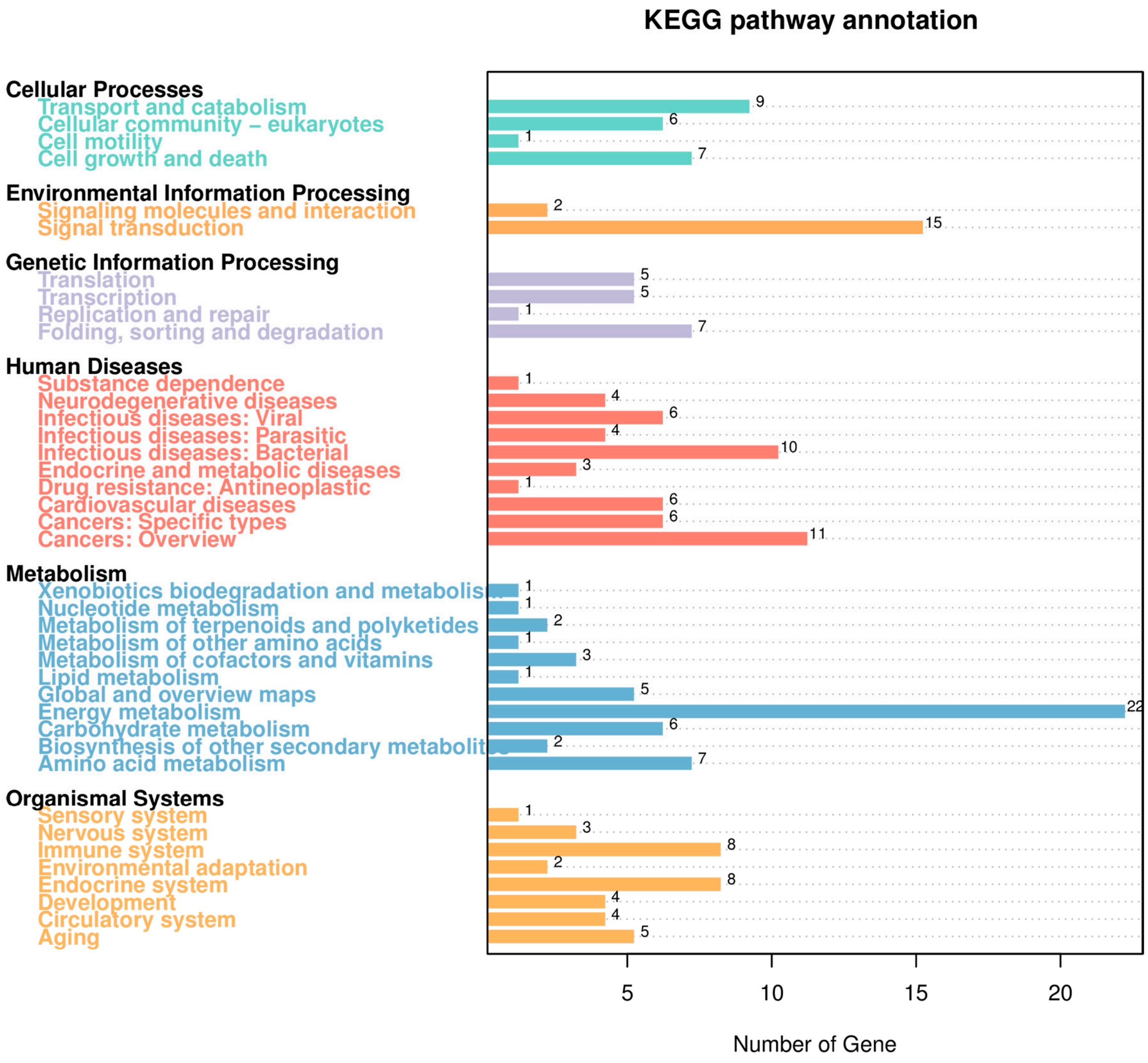

Figure S23. KEGG metabolic pathway classifications of Symbiodiniaceae genes in *P. verrucosa*. On the left are the different KEGG categories, including cellular processes, environmental information processing, genetic information processing, human diseases, metabolism and organismal systems. On the right is the number of their corresponding genes.

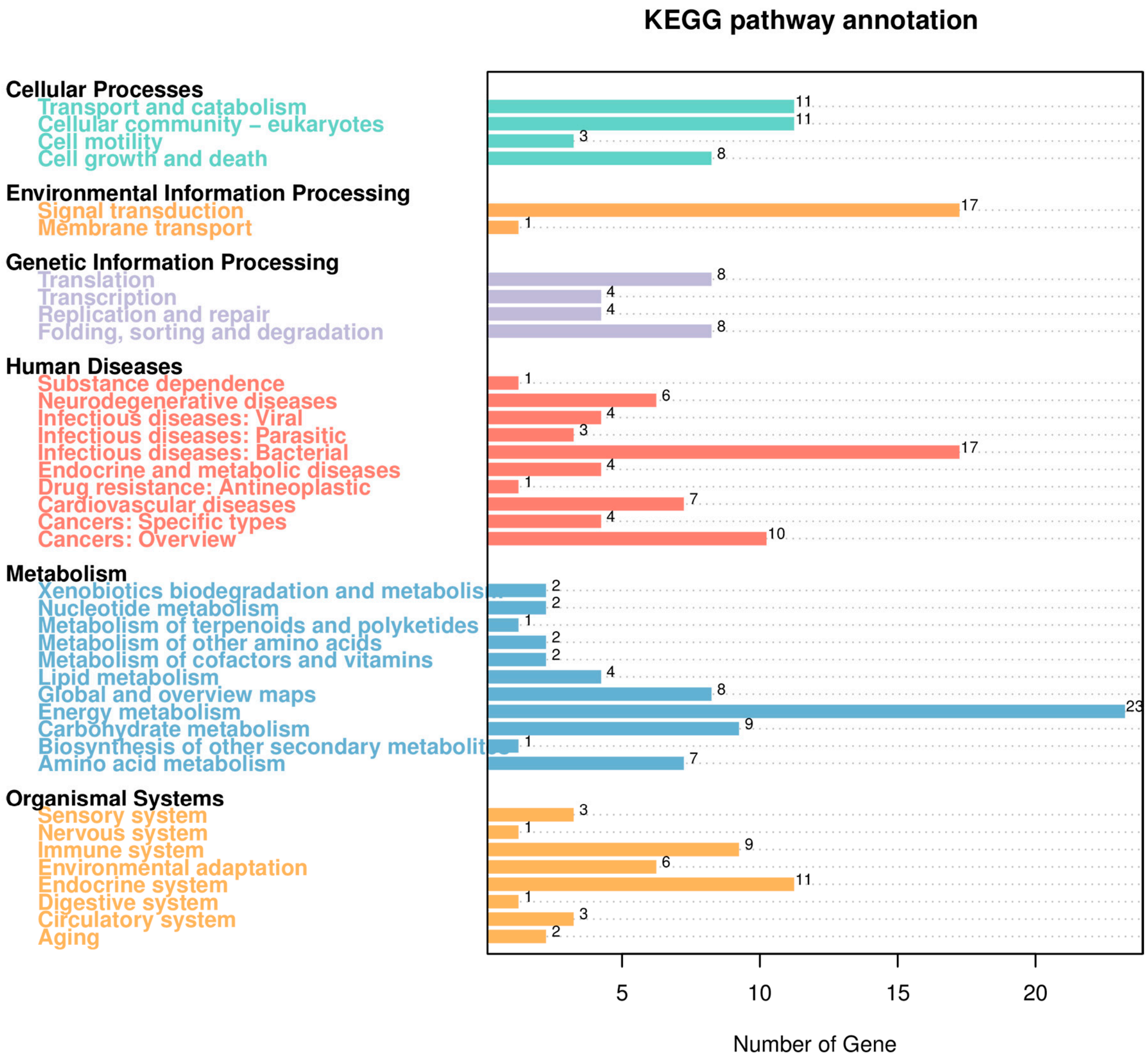

Figure S24. KEGG metabolic pathway classifications of Symbiodiniaceae genes in *A. muricata*. On the left are the different KEGG categories, including cellular processes, environmental information processing, genetic information processing, human diseases, metabolism and organismal systems. On the right is the number of their corresponding genes.

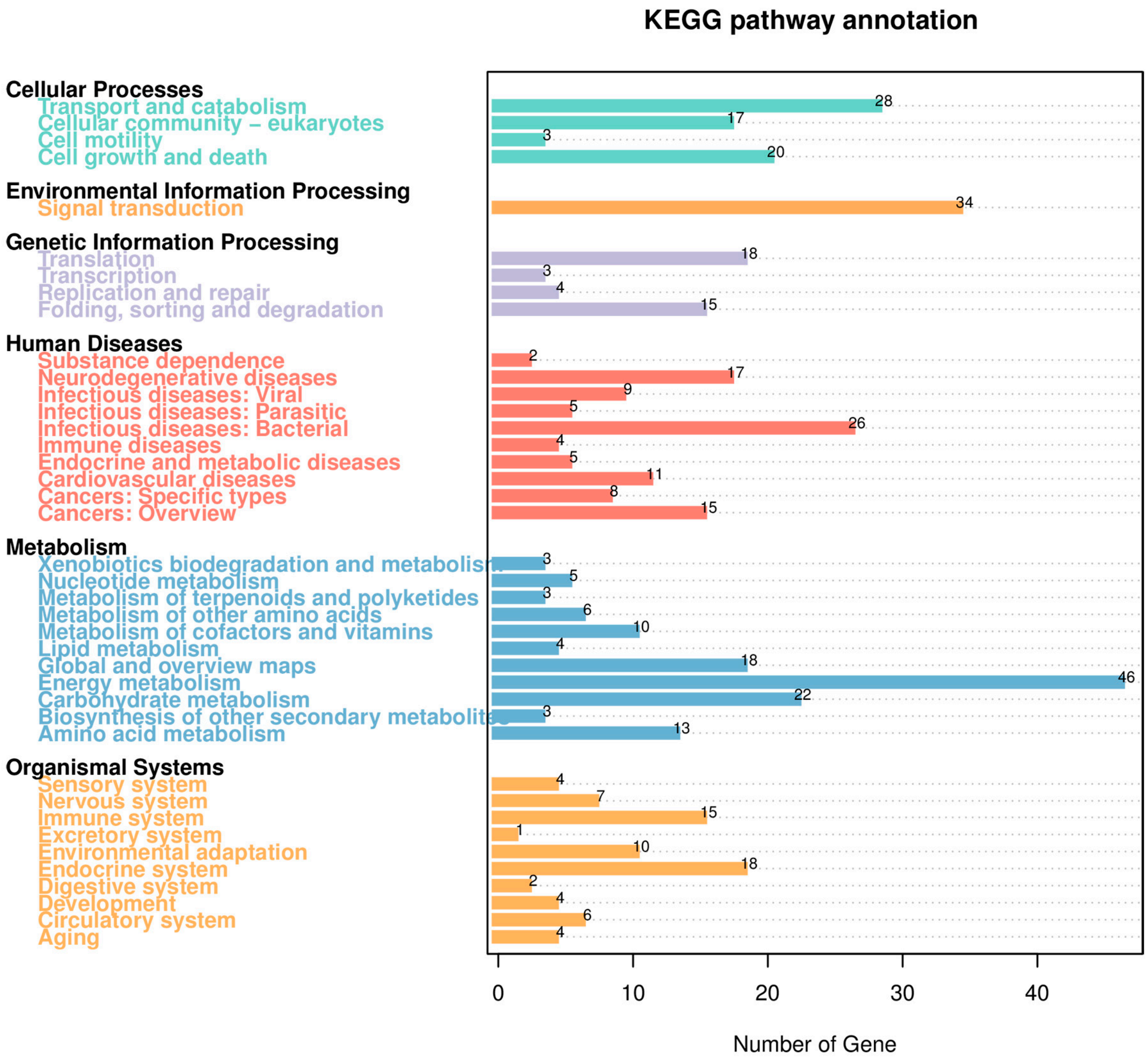

Figure S25. KEGG metabolic pathway classifications of Symbiodiniaceae genes in *M. foliosa*. On the left are the different KEGG categories, including cellular processes, environmental information processing, genetic information processing, human diseases, metabolism and organismal systems. On the right is the number of their corresponding genes.

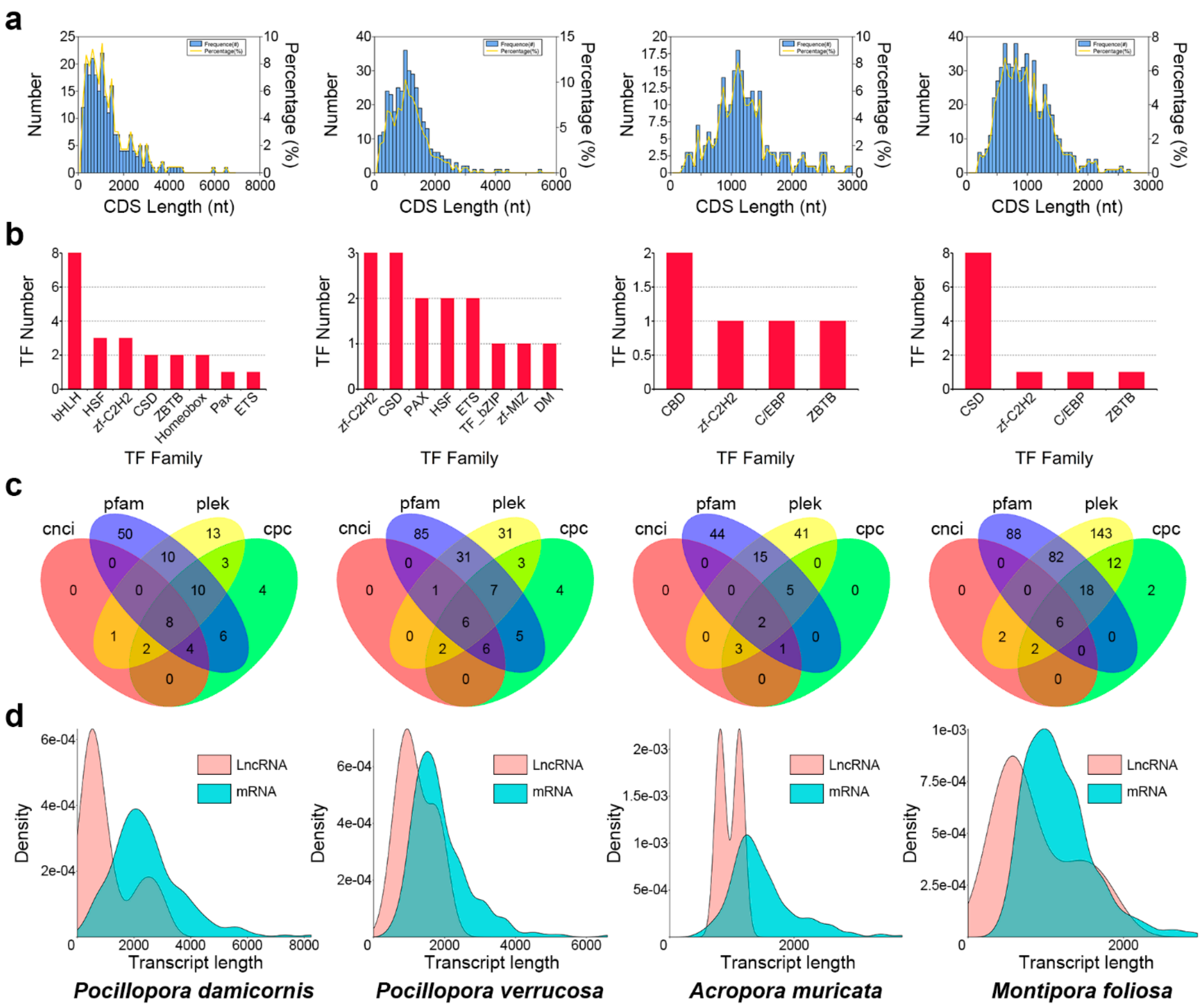

Figure S26. Summary of Symbiodiniaceae gene structural analysis. **a.** CDS length distribution. The horizontal axis represents the length of the predicted CDS, the vertical axis represents the number (blue bar chart) and percentage (yellow curve chart) of transcripts of the CDS. **b**. Predicted TF family. The horizontal axis represents the top 8 or 4 predicted transcription factor families, and the vertical axis represents the number of them. **c**. Venn plots of predicted lncRNA. The sum of the numbers in each large circle represents the number of lncRNA predicted by one of CNCI, PLEK, CPC2 and Pfam databases, and the overlapping circles indicate the number of lncRNA predicted by these two or more databases simultaneously. **d**. LncRNA and mRNA length distribution comparison. The horizontal axis is the length of transcripts, the vertical axis is their density of distribution.

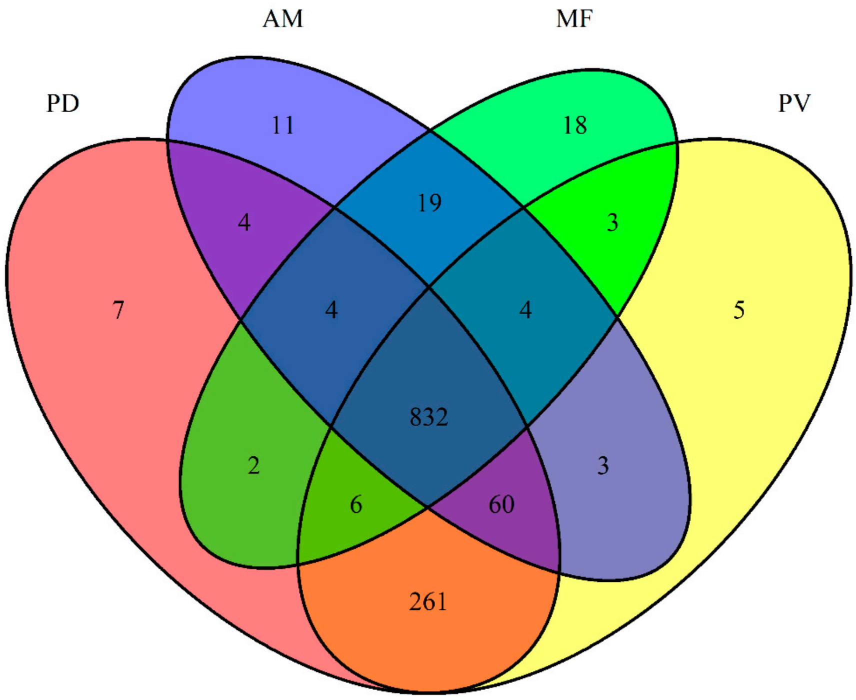

Figure S27. Venn plots of statistics on the number of Symbiodiniaceae sequences expressed in four reef-building corals. The sum of the numbers in each large circle represents the number of Symbiodiniaceae sequences expressed in one of the corals, and the overlapping circles indicate the number of Symbiodiniaceae sequences expressed in these two or more corals simultaneously. PD: *P. damicornis*; PV: *P. verrucosa*; AM: *A. muricata*; and MF: *M. foliosa*

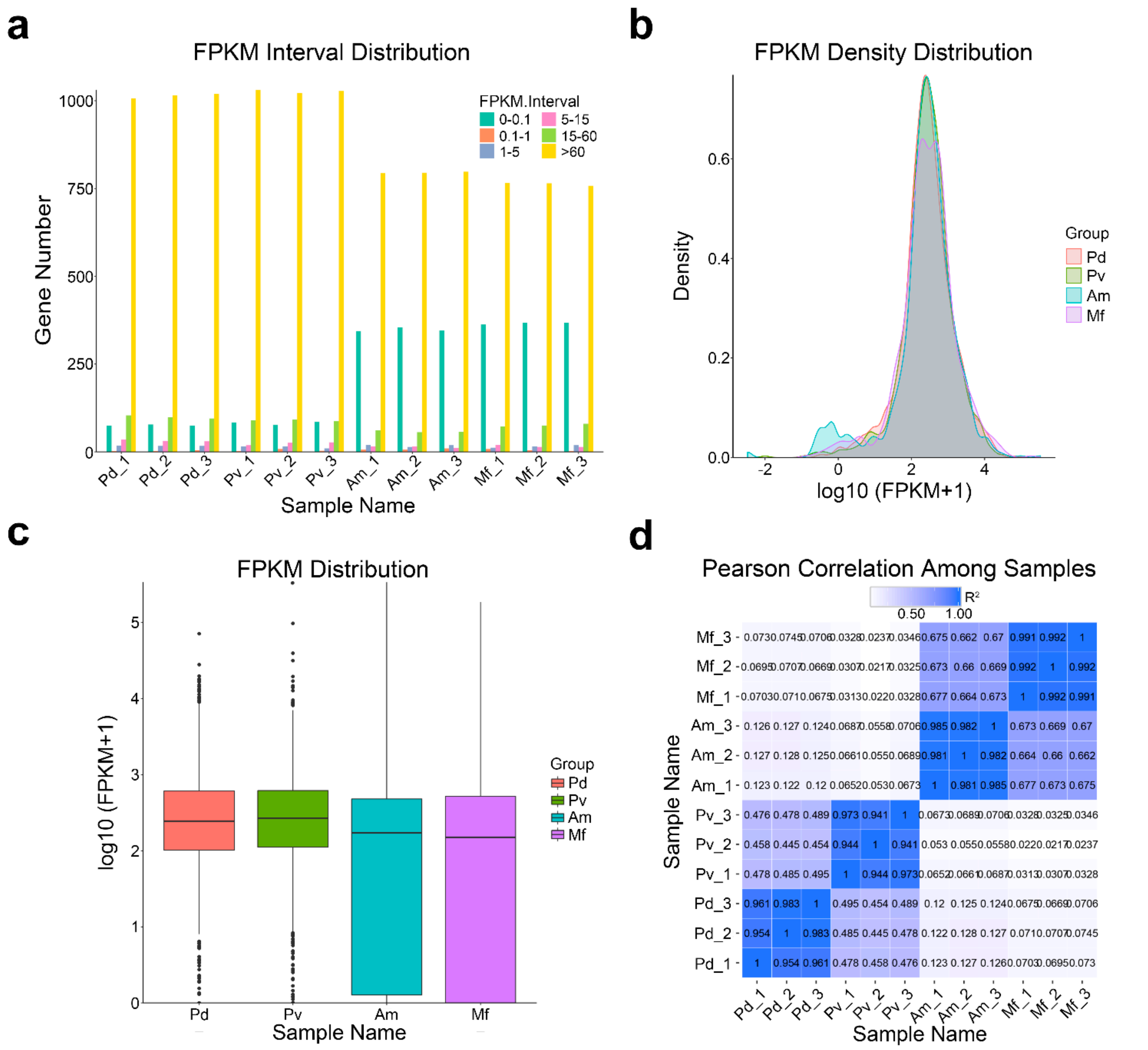

Figure S28. Summary of the Symbiodiniaceae gene expression level analysis. **a**. FPKM interval distribution. The horizontal axis shows the sample name, different colours represent FPKM intervals, and the vertical axis represents the number of genes in each interval. **b**. FPKM density distribution. The horizontal axis represents the log10 (FPKM+1) values, and the vertical axis represents the density of genes with different expression levels. **c**. FPKM box plot. The horizontal axis shows the sample name, and the vertical axis represents the log10 (FPKM+1) values. Each box plot shows five statistical values, including the maximum, upper quartile, median, lower quartile and minimum, from top to bottom. **d**. Pearson correlation among samples. The closer the value is to 1, the better the correlation. Pd: *P. damicornis*; Pv: *P. verrucosa*; Am: *A. muricata*; and Mf: *M. foliosa*.

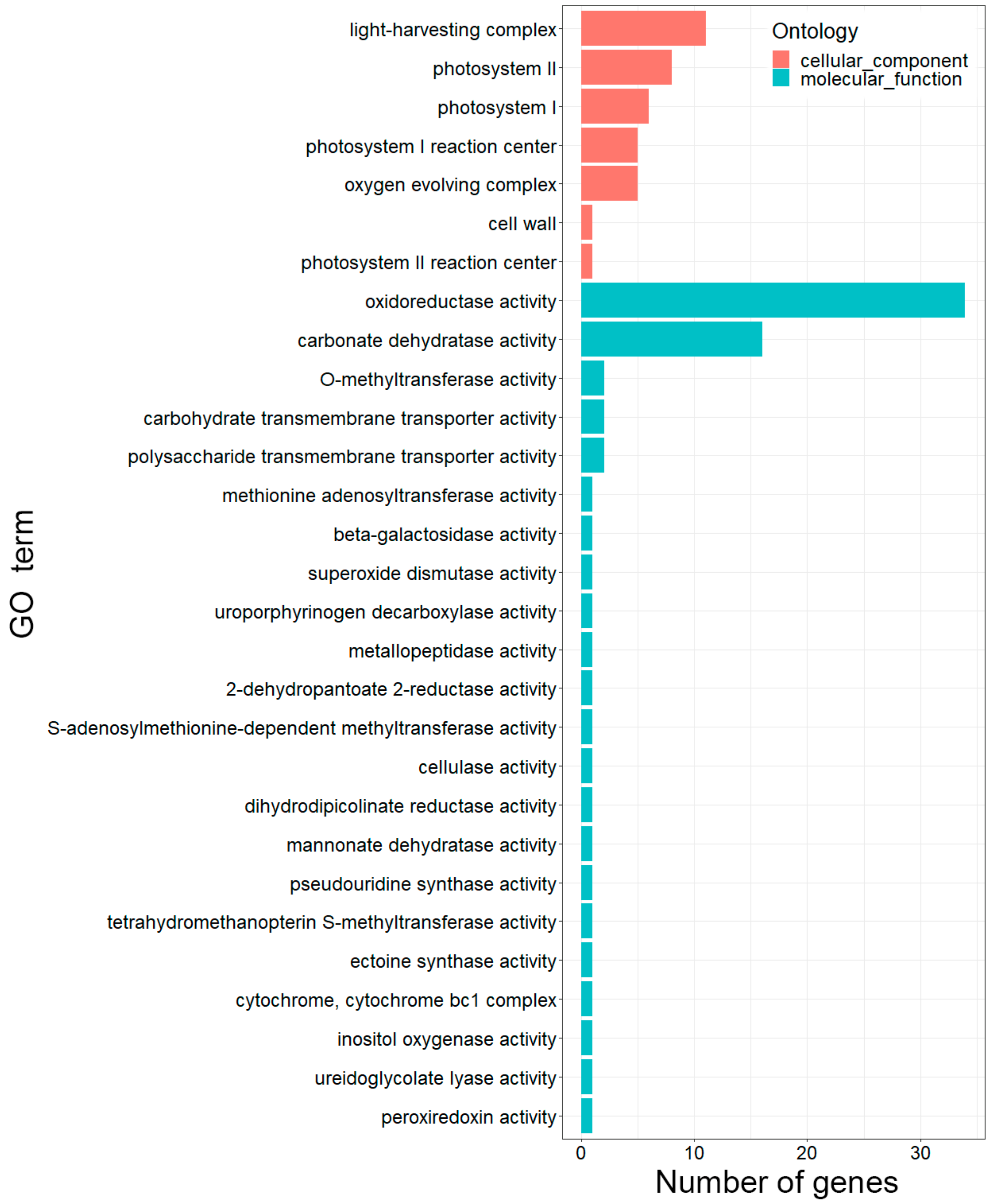

Figure S29. Bar graph of coexpressed Symbiodiniaceae sequences based on GO molecular functions and cellular components categories. The vertical axis represents the GO terms (orange represents molecular functions and blue-green represents cellular components), and the horizontal axis represents the number of transcripts annotated to the terms (including sub terms of the terms).

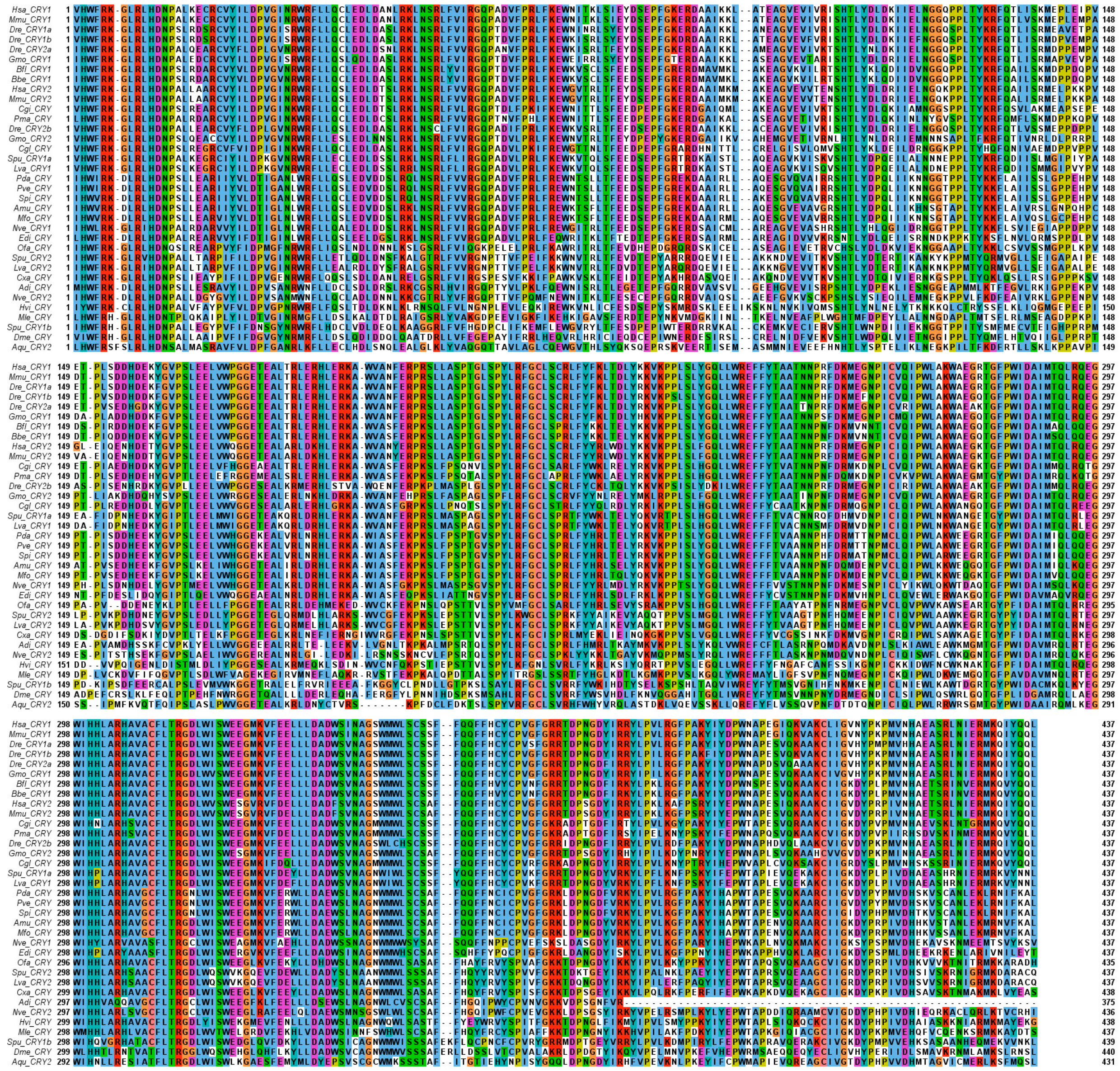

Figure S30. Conserved domain of *cry1* or *2* genes among different species.

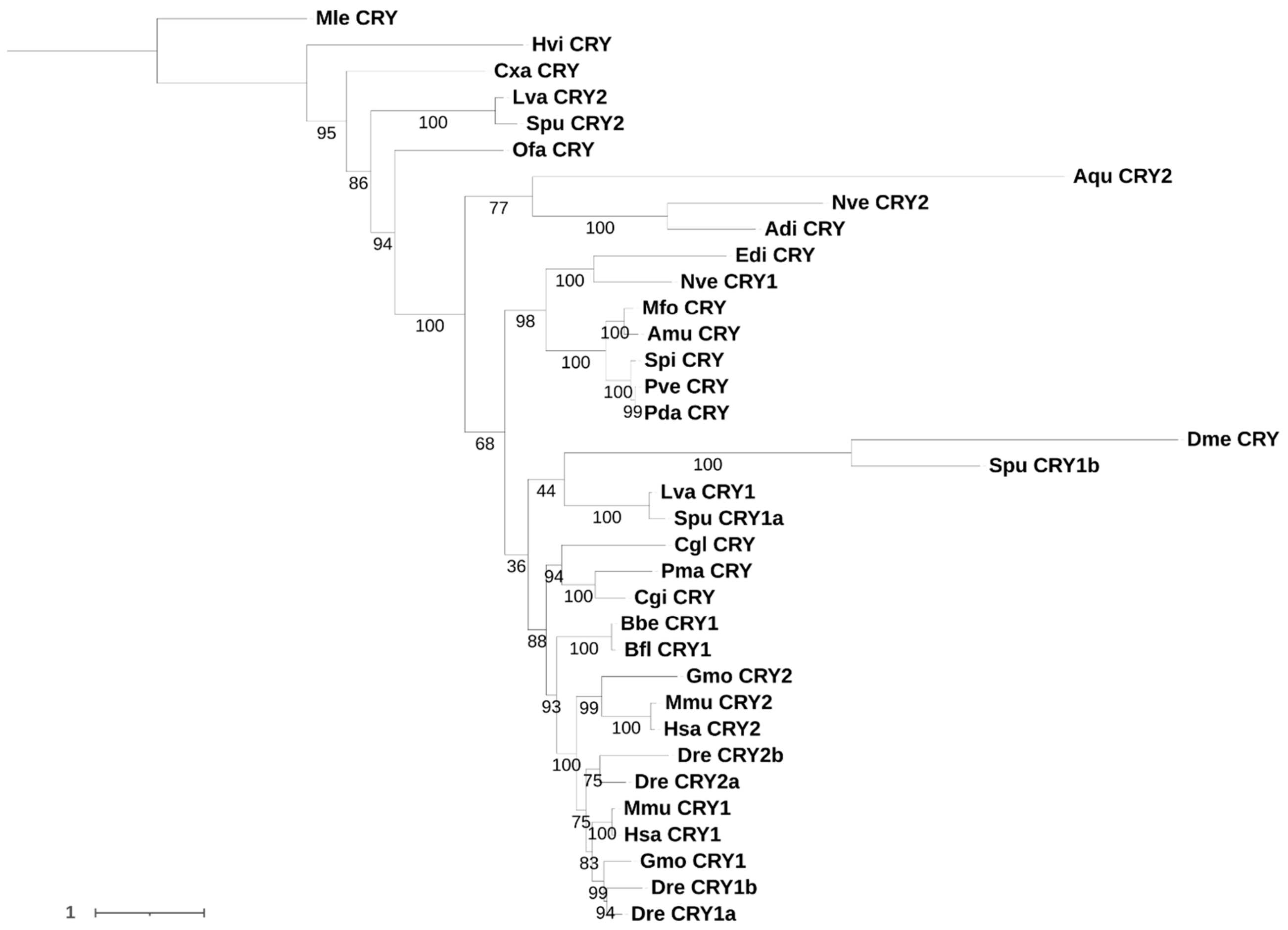

Figure S31. Phylogenetic tree of *cry1* or *2* genes based on the maximum likelihood method with best-fit model selection. Branch lengths are optimized by maximum likelihood on original alignment and the numbers are bootstrap supports (%).

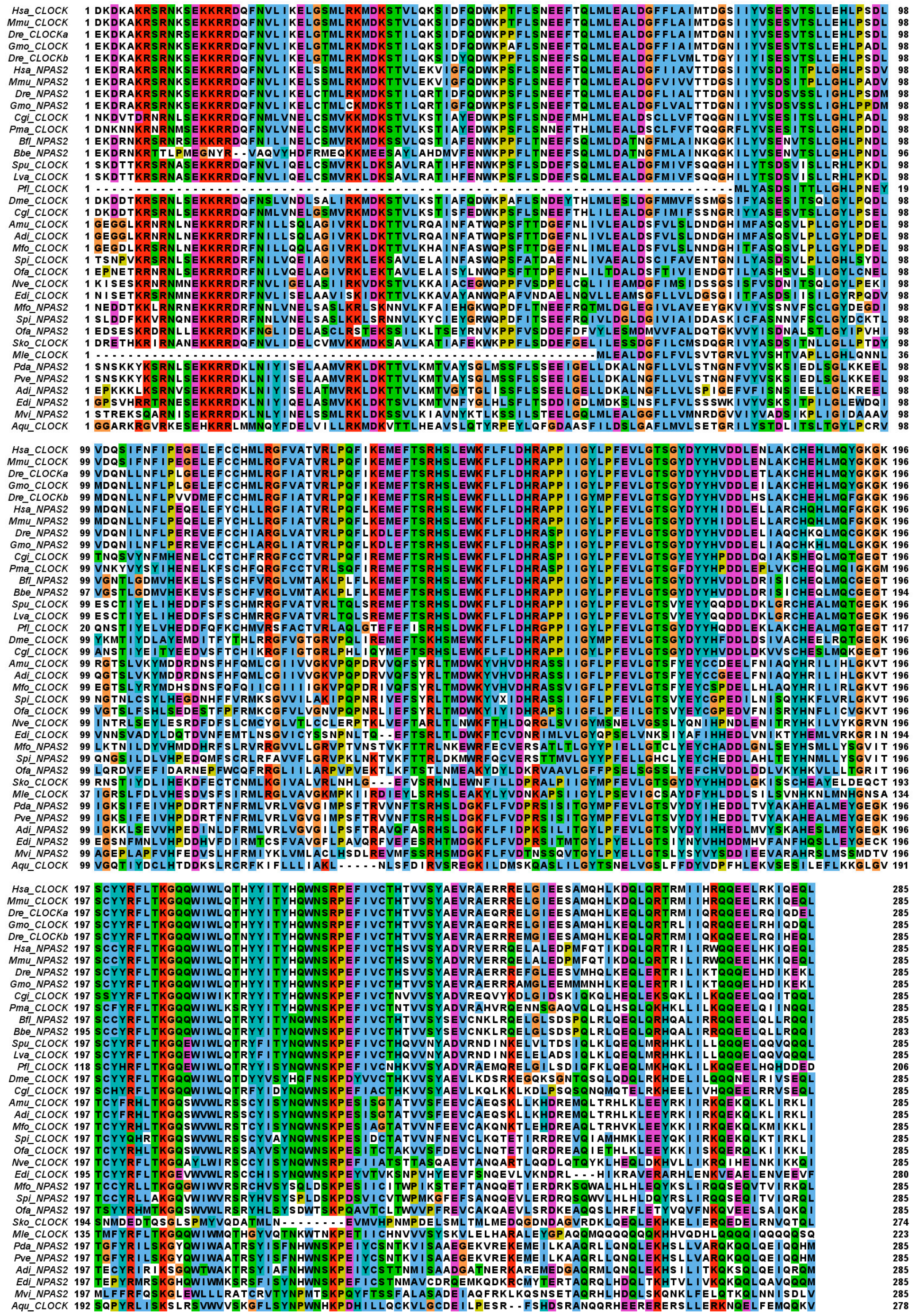

Figure S32. Conserved domain of *Clock* or *Npas2* genes among different species.

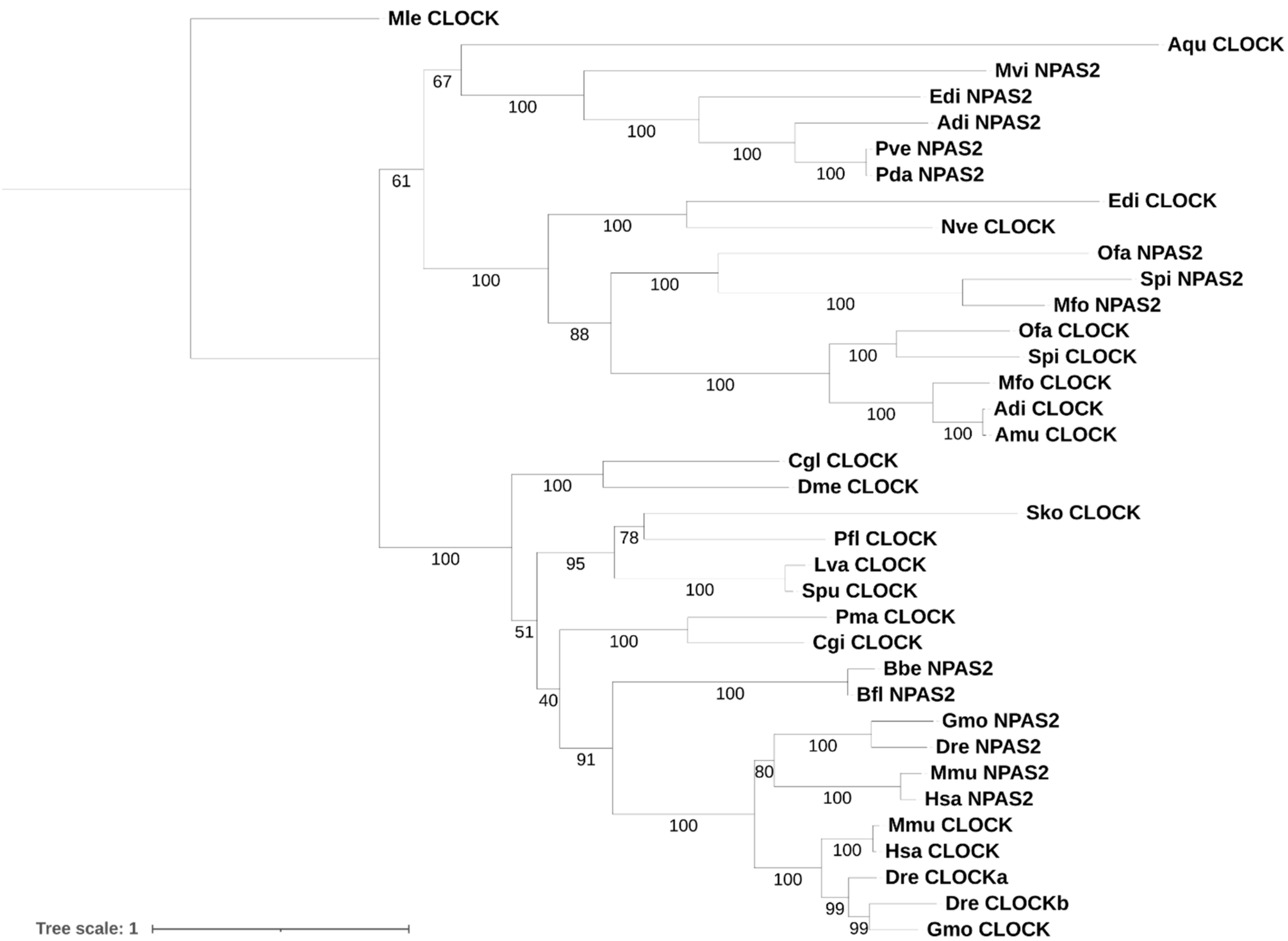

Figure S33. Phylogenetic tree of *Clock* or *Npas2* genes based on the maximum likelihood method with best-fit model selection. Branch lengths are optimized by maximum likelihood on original alignment and the numbers are bootstrap supports (%).

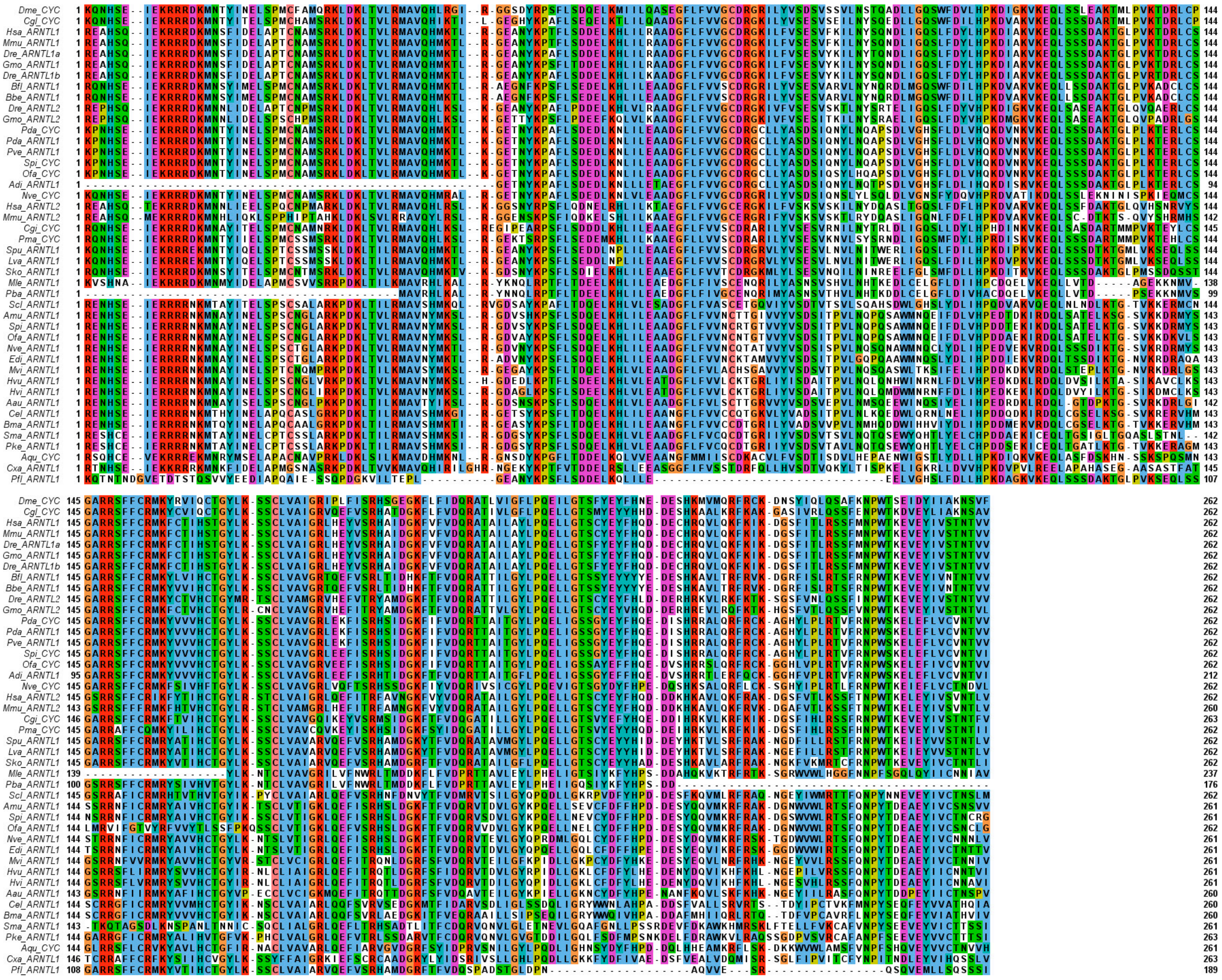

Figure S34. Conserved domain of *cyc* or *Arntl* genes among different species.

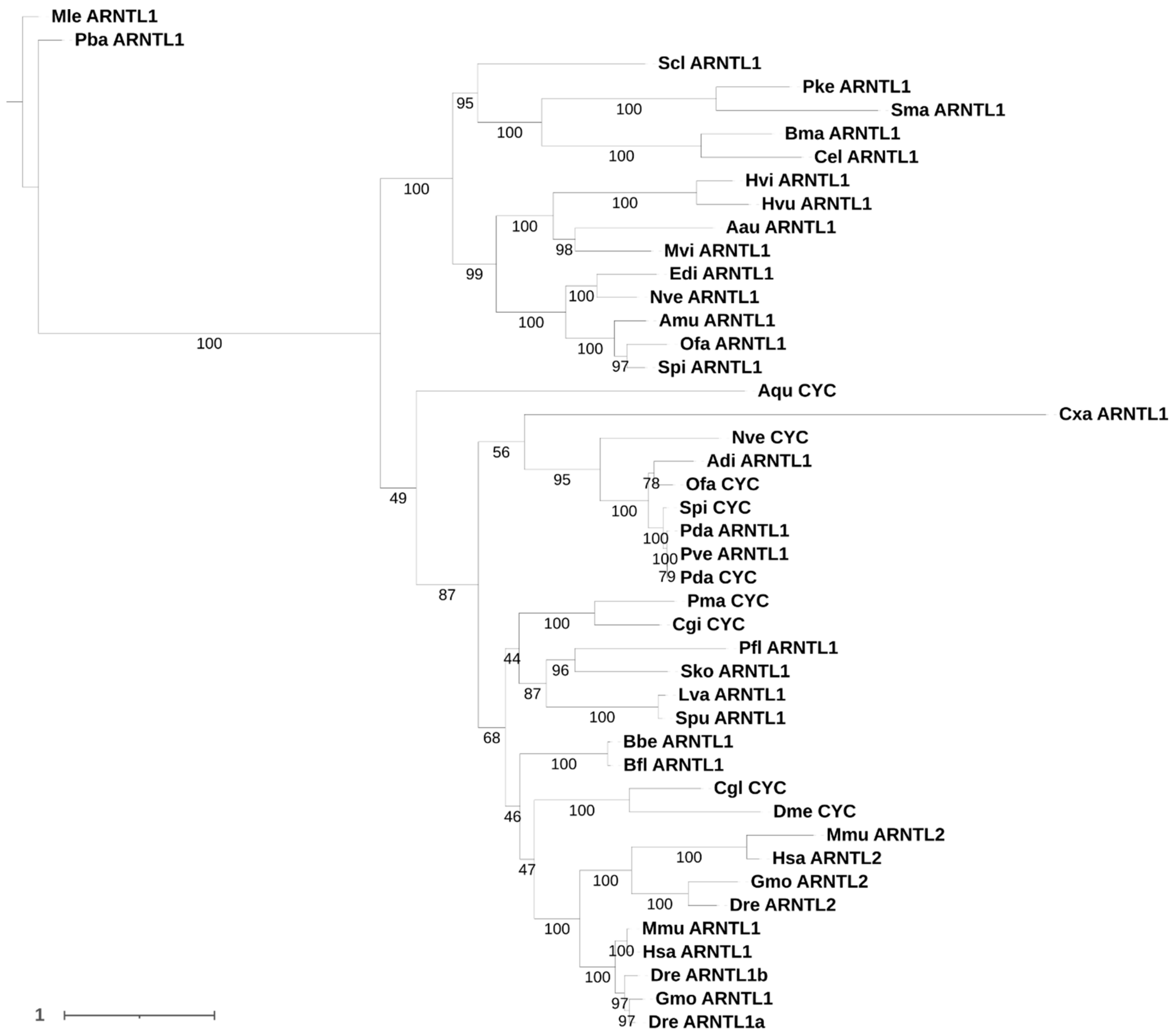

Figure S35. Phylogenetic tree of *cyc* or *Arntl* genes based on maximum likelihood method with best-fit model selection. Branch lengths are optimized by maximum likelihood on original alignment and the numbers are bootstrap supports (%).

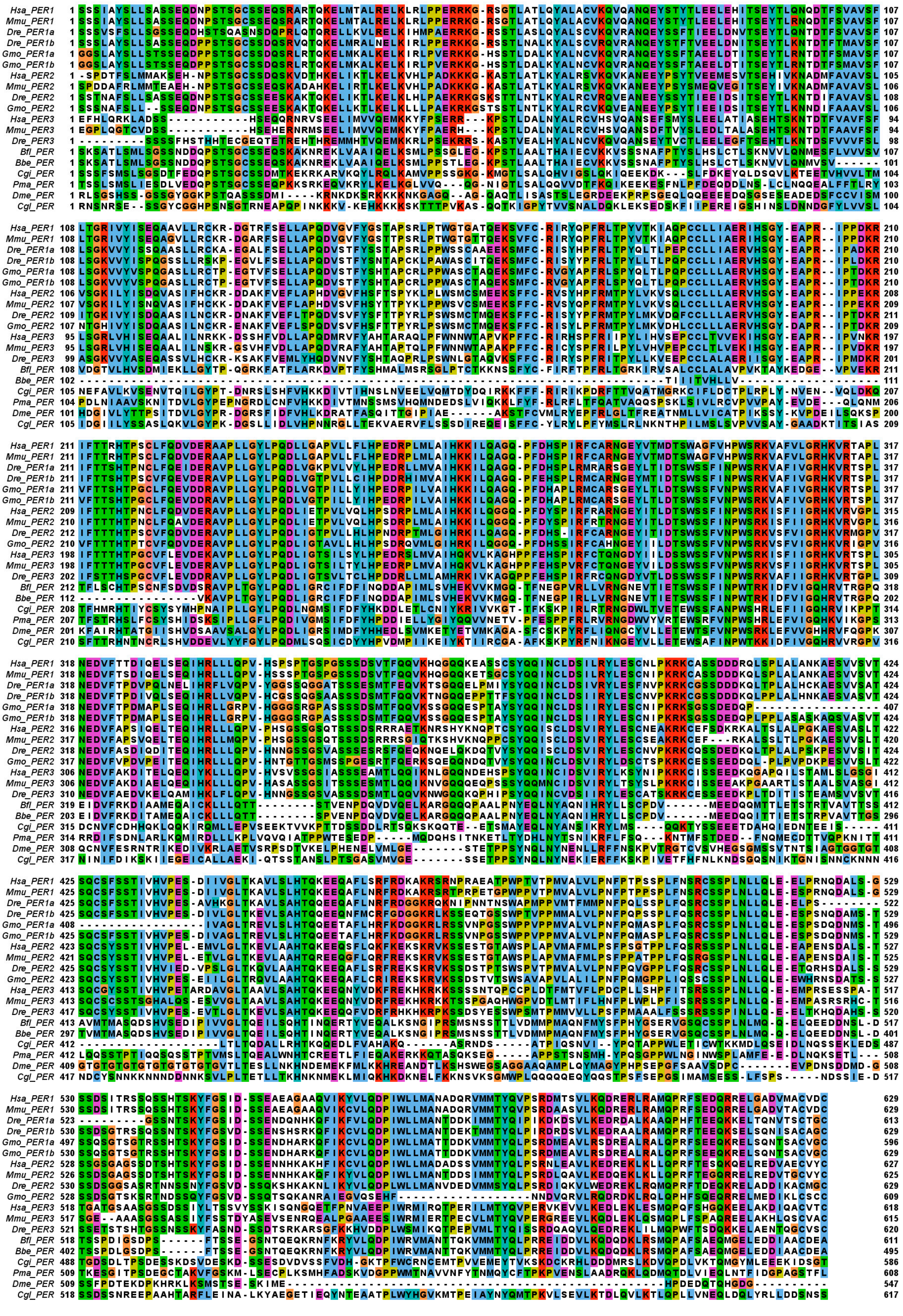

Figure S36. Conserved domain of *per* genes among different species.

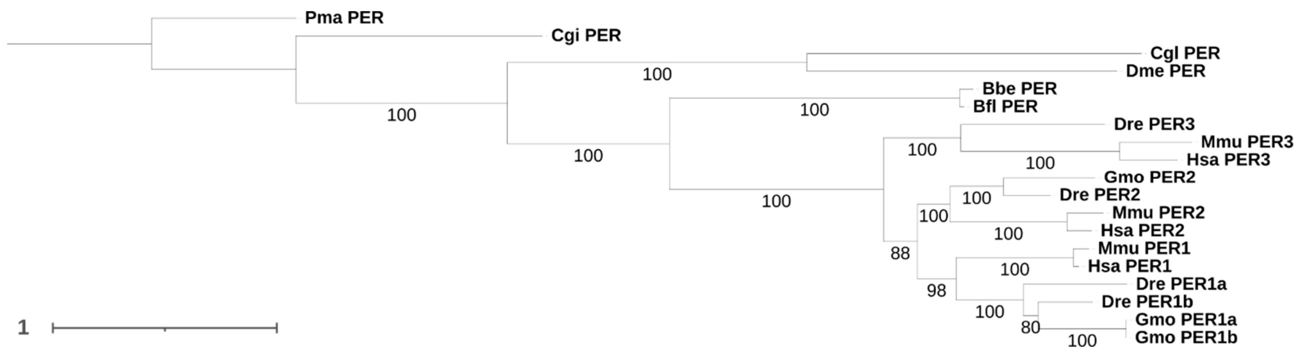

Figure S37. Phylogenetic tree of *per* genes based on the maximum likelihood method with best-fit model selection. Branch lengths are optimized by maximum likelihood on original alignment and the numbers are bootstrap supports (%).

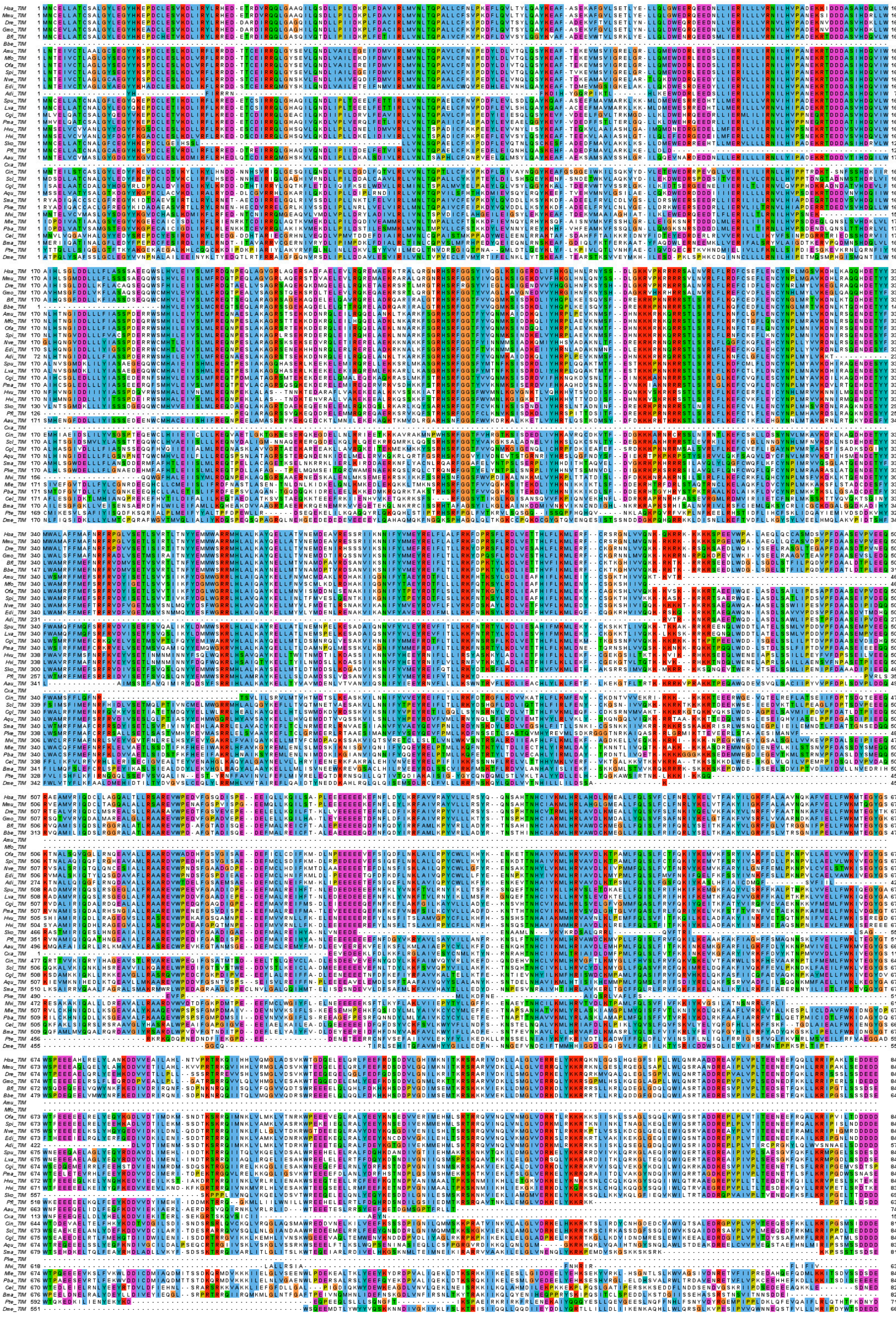

Figure S38. Conserved domain of *tim* genes among different species.
